# Bio‐Inspired Self‐Assembly of Enzyme‐Micelle Systems for Semi‐Artificial Photosynthesis

**DOI:** 10.1002/anie.202424222

**Published:** 2025-03-03

**Authors:** Yongpeng Liu, Santiago Rodríguez‐Jiménez, Hongwei Song, Andrea Pannwitz, Dongseok Kim, Ana M. Coito, Rita R. Manuel, Sophie Webb, Lin Su, Shannon A. Bonke, Ross D. Milton, Inês A. C. Pereira, Sylvestre Bonnet, Leif Hammarström, Erwin Reisner

**Affiliations:** ^1^ Yusuf Hamied Department of Chemistry University of Cambridge Lensfield Road Cambridge CB2 1EW UK; ^2^ Department of Chemistry – Angstrom Laboratory Uppsala University 751 20 Uppsala Sweden; ^3^ Leiden Institute of Chemistry Leiden University 2333 CC Leiden, The Netherlands; ^4^ Instituto de Tecnologia Química e Biológica António Xavier (ITQB NOVA) Universidade NOVA de Lisboa 2780-157 Oeiras Portugal; ^5^ Department of Inorganic and Analytical Chemistry University of Geneva 1211 Geneva 4 Switzerland; ^6^ National Centre of Competence in Research (NCCR) Catalysis University of Geneva 1211 Geneva 4 Switzerland

**Keywords:** photocatalysis, surfactants, micelles, hydrogenases, formate dehydrogenases

## Abstract

Supramolecular surfactants provide a versatile platform to construct systems for solar fuel synthesis, for example via the self‐assembly of amphiphilic photosensitizers and catalysts into diverse supramolecular structures. However, the utilization of amphiphilic photosensitizers in solar fuel production has predominantly focused on yielding gaseous products, such as molecular hydrogen (H_2_), carbon monoxide (CO), and methane (CH_4_) with turnover numbers (TONs) of synthetic catalysts typically in the range of hundreds to thousands. Inspired by biological lipid‐protein interactions, we present herein a bio‐hybrid assembly strategy that utilizes photosensitizers as surfactants to form micellar scaffolds that interface with enzymes, namely hydrogenases and formate dehydrogenases, for semi‐artificial photosynthesis. Specifically, surfactants with a tris(2,2’‐bipyridine)ruthenium(II) head group provide high photocatalytic activity upon association with the enzymes as their positively charged [Ru(bpy)_3_]^2+^ complex electrostatically interacts with the enzymes favorably to enable direct electron transfer at the micelle‐enzyme interface. Time‐resolved absorption and emission spectroscopy support the beneficial charge carrier dynamics of the reductively quenched [Ru(bpy)_3_]^+^ species when the enzymes are introduced in the micellar solution. Thus, a biohybrid concept is introduced for solar fuel synthesis using a biomimetic enzyme‐micellar system, providing also a platform for other photocatalytic transformations using enzymes in the future.

## Graphical abstract

This study introduces a bio‐hybrid assembly inspired by lipid‐protein interactions in nature by utilizing photosensitized surfactants that self‐assemble into light‐absorbing micellar scaffolds capable of interfacing with hydrogenases and formate dehydrogenases. This semi‐artificial approach enabled benchmark turnover numbers for solar H_2_ production and CO_2_‐to‐formate conversion, establishing a versatile platform for future photocatalytic transformations.

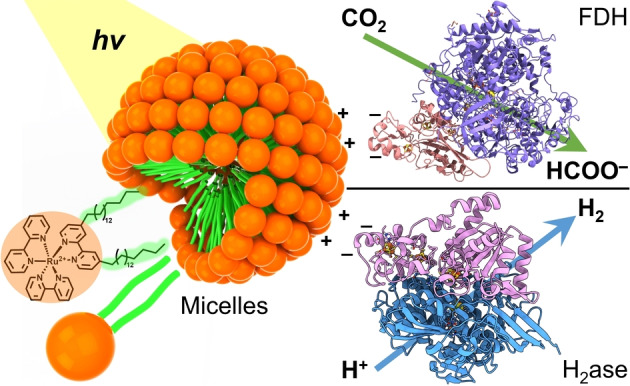

## Introduction

Semi‐artificial photosynthesis brings together biological and synthetic components capable of synergistically harnessing solar energy and transforming it into clean chemical fuels.^[1]^ Solar hydrogen (H_2_) generation and carbon dioxide (CO_2_) reduction have the potential to produce carbon‐free energy carriers.^[2]^ While synthetic electrocatalysts are traditionally paired with dyes in artificial photosynthesis, their turnover numbers (TONs) and selectivity for CO_2_ reduction are often low. In contrast, enzymes such as hydrogenase (H_2_ase) and formate dehydrogenase (FDH) have evolved in nature to catalyze the interconversion of protons and electrons to H_2_ as well as CO_2_, protons and electrons to formate, respectively.^[3]^ The strategy of activating enzymes with sunlight for semi‐artificial photosynthesis combines the strength of enzymes (efficiency and selectivity) for catalysis^[4]^ with synthetic materials (tunability and scalability) for light absorption,^[5]^ offering great potential to innovate new approaches for light‐driven chemistry.^[6]^

Biological membranes, made of lipid bilayers, are crucial for the functioning of cells and organisms, by enabling reaction compartmentalization, selective transport and enabling specific proton‐protein interactions.^[7]^ The amphipathic nature of lipid molecules therefore offers unique properties for maintaining the physiological function of cells in all forms of life.^[8]^ Similar to a lipid, a surfactant molecule consists of a hydrophilic head and hydrophobic tails, which grants its amphiphilicity. At a surfactant concentration above the critical micelle concentration (CMC), surfactants are self‐assembled in the bulk solution forming supramolecular structures such as bilayers, micelles, liposomes, and giant vesicles.^[9]^

The self‐assembly of synthetic amphiphilic photosensitizers and surfactant into diverse structures such as micelles and liposomes has already been established as a versatile supramolecular platform for hosting co‐catalysts, providing a design strategy to assemble systems for solar fuel synthesis.[^10^, ^11^] Surfactants for photochemical H_2_ evolution were already reported almost 50 years ago, using dioctadecyl‐ and dihydrocholesteryl‐modified [Ru(bpy)_3_]^2+^.[^12^, ^13^] More recently, amphiphilic photosensitizers and molecular catalysts were embedded into surfactant membranes for light‐driven H_2_ evolution and CO_2_ reduction. Notably, the group of Wu, König and Reek have demonstrated the incorporation of [Ru(bpy)_3_]^2+^ surfactants and [FeFe]‐H_2_ase mimics for photocatalytic H_2_ production.[^14^, ^15^, ^16^] While their systems achieved direct electron transfer (DET) for the H_2_ evolution reaction (HER), the TONs remained low (<600).[^14^, ^15^, ^16^, ^17^]

Light‐driven H_2_ evolution by H_2_ase using self‐assembled surfactants has been reported, for example, using [Ru(bpy)_3_]^2+^ surfactants in reversed micelles to isolate H_2_ase and the redox mediator methyl viologen from an organic solvent mixture.^[18]^ Zinc tetraphenylporphyrin (ZnTPP) as photosensitizer in vesicles and micelles was also used to activate H_2_ase in aqueous solution containing methyl viologen.[^19^, ^20^] However, previous systems only achieved low TONs for the H_2_ase and relied on methyl viologen as the electron shuttle and direct electron transfer from the dye to the enzyme has thereby not been achieved. The instability, toxicity and susceptibility to back‐reactions severely limits the applicability of such systems using the redox mediator methyl viologen.^[21]^

Surfactants have also been used for the photocatalytic reduction of CO_2_, predominantly in conjunction with molecular catalysts for carbon monoxide (CO) and methane (CH_4_) production, yielding low TONs. For instance, He and co‐workers utilized micellar rhenium (Re) catalysts to achieve visible‐light‐driven CO_2_ reduction to CO with a TON up to 110.^[22]^ Similarly, Murata and co‐workers constructed vesicles containing molecular Ru and Re catalysts for CO_2_ to CO conversion, attaining a TON of 190.^[23]^ Recently, our laboratory employed a Ru liposome and a cobalt(II) porphyrin surfactant as catalyst, resulting in a TON of 1,456 for light‐driven CO_2_‐to‐CO conversion.^[24]^ This cobalt(II) porphyrin catalyst for solar CO_2_ reduction to CO has subsequently also been used in a micellar self‐assembly system with a surfactant Ru dye with holistic optimization of performance using machine learning algorithms, resulting in a TON of 422.^[25]^ Tian and colleagues generated a spherical chromatophore micelle made of self‐assembled amphiphilic Zn porphyrin photosensitizers with an homogeneous Co catalyst, achieving a TON of 6,600 for solar CO_2_‐to‐methane conversion.^[26]^ To the best of our knowledge, the direct interfacial electron transfer at a self‐assembled surfactant‐enzyme biohybrid system or the photocatalytic reduction of CO_2_ to liquid products such as formate has not yet been achieved.

Inspired by the biological lipid‐protein association in vivo,^[8]^ and electrostatically assembled light absorber‐enzyme hybrids in vitro,[^27^, ^28^] we developed in this work a semi‐artificial photosynthetic assembly consisting of micelle‐forming amphiphilic photosensitizers and enzymes for direct and efficient solar H_2_ and formate production. A [Ru(bpy)_3_]^2+^ hydrophilic head with alkyl tails containing different carbon chain lengths have been employed, together with H_2_ase and FDH. Photocatalytic activities for HER and CO_2_ reduction have been achieved through direct photo‐induced Ru‐surfactant‐enzyme interfacial electron transfer, and solar CO_2_ reduction to formate is demonstrated thanks to interfacial engineering of the semi‐artificial micelles. The charge carrier dynamics and effects of carbon chain length have been revealed by nanosecond transient absorption spectroscopy (TAS). Mechanistic studies indicate that a strong association between micelles and enzymes is a prerequisite for direct electron transfer.

## Results and Discussion

### Synthesis and Characterization of Ru‐Micelles

**Figure 1:**
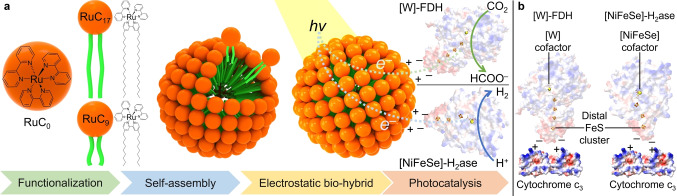
(a) Schematic illustration of RuC_0_, RuC_9_ and RuC_17_ dyes (charges and counter‐ions omitted for clarity), self‐assembly of micelles, electrostatic assembly of bio‐hybrids with *Dv*H [W]‐FDH (PDB: 6sdv) and *Dv*H [NiFeSe]‐H_2_ase (PDB: 5jsh) and solar CO_2_ reduction to formate and solar proton reduction to H_2_ in vitro, enabled by direct electron transfer at the micelle‐enzyme interface. (b) The in vivo electrostatic interaction between *Dv*H [W]‐FDH (PDB: 6sdv) and cytochrome c_3_ (PDB: 2cth), and between *Dv*H [NiFeSe]‐H_2_ase (PDB: 5jsh) and cytochrome c_3_ (PDB: 2cth), illustrating the biomimetic feature of the RuC_*n*_|enzyme biohybrids.

Direct electron transfer relies on constructing a productive interfacial contact between the light absorber and enzyme. Even covalently linking a Ru dye to a H_2_ase can result in an unproductive interface, still requiring methyl viologen for charge transfer.[^29^, ^30^] To overcome this challenge, semiconductors such as TiO_2_ have been employed to act as heterogeneous electron relays to photoreduce enzymes (H_2_ase and FDH) with immobilized [Ru(bpy)_3_]^2+^ dyes.[^31^, ^32^] Inspired by ubiquitous enzyme‐membrane interactions in vivo, we deployed an amphiphilic [Ru(bpy)_3_]^2+^ with alkyl chains to form lipid‐like RuC_*n*_ photosensitizers, where the number of carbon atoms in the alkyl chain (*n*) is either 9 or 17 (Figure 1a). As enzymes, we selected the metalloproteins [NiFeSe]‐H_2_ase and [W]‐FDH from *Desulfovibrio vulgaris* Hildenborough (*Dv*H),[^33^, ^34^] as well as [FeFe]‐H_2_ase from *Clostridium pasteurianum* (*Cp*I; see Supporting Information for isolation and purification of enzymes).^[35]^

Previous works confirmed that no lipid components remain after enzyme purification, avoiding potential interference on the photocatalytic experiment.[^33^, ^34^, ^35^] These enzymes display high electrocatalytic activity and differently charged regions close to the electron entry point to interact with RuC_*n*_ surfactants (Figure 1b).

The synthesis and characterization of the Ru‐surfactants are described in the Supporting Information (Figure S1–S9), and are consistent with previous reports.[^24^, ^25^, ^36^] The amphiphilic RuC_*n*_ surfactants self‐assemble into micellar structures with a positively charged surface that interfaces with H_2_ase and FDH. The resulting RuC_*n*_|enzyme biohybrids allow for solar‐driven proton reduction to H_2_ and CO_2_ reduction to formate, respectively (Figure 1a). The micelle aggregation number which describes the average number of surfactants required to form one spherical micelle,^[37]^ is predominantly determined by the hydrophobic tail length and can be estimated by a predictive molecular thermodynamic approach.^[38]^ The micelle aggregation number for RuC_9_ and RuC_17_ surfactants is estimated as 33 and 104, respectively (see Supporting Information for calculations). Note that the presence of MOPS buffer in the solution can lead to a higher micelle aggregation number due to the screened electrostatic repulsion.[^37^, ^39^] A positive zeta potential value of 6.3±1.2 mV for RuC_9_ and 21.6±3.5 mV for RuC_17_ is observed, which is attributed to the positively charged hydrophilic [Ru(bpy)_3_]^2+^ head. The zeta potential of RuC_17_ is approximately three times higher than that of RuC_9_, which is close to the ratio between their micelle aggregation numbers.

### Photocatalysis using Micelle‐H_2_ase Bio‐Hybrid

Photo‐induced electron delivery from a light absorber to an enzyme requires an ‘electroactive’ orientation of the latter.^[40]^ Interfacial electron transfer in *Dv*H [NiFeSe]‐H_2_ase occurs through a distal FeS cluster near the protein surface. The surrounding protein region of this distal FeS cluster is negatively charged, which therefore interacts electrostatically with positively charged residues, such as those surrounding the heme co‐factor of cytochrome *c*_3_ for in vivo catalysis (Figure 1b).[^41^, ^42^] Inspired by this in vivo electrostatic interactions, we interface [NiFeSe]‐H_2_ase with positively charged RuC_*n*_ surfactants to achieve a productive electrostatic in vitro assembly. The resulting photocatalytic RuC_*n*_|[NiFeSe]‐H_2_ase systems also contain sodium ascorbate (NaHAsc, 0.1 M) as the sacrificial electron donor, and MOPS buffer (0.1 M, pH 7) in an anaerobic photoreactor with a headspace of 4.5 mL. Under simulated solar (AM 1.5G) irradiation, the bio‐hybrids produced H_2_ (quantified by gas chromatography) in the absence of an electron mediator, supporting DET between amphiphilic photosensitizers and the H_2_ase.

**Figure 2:**
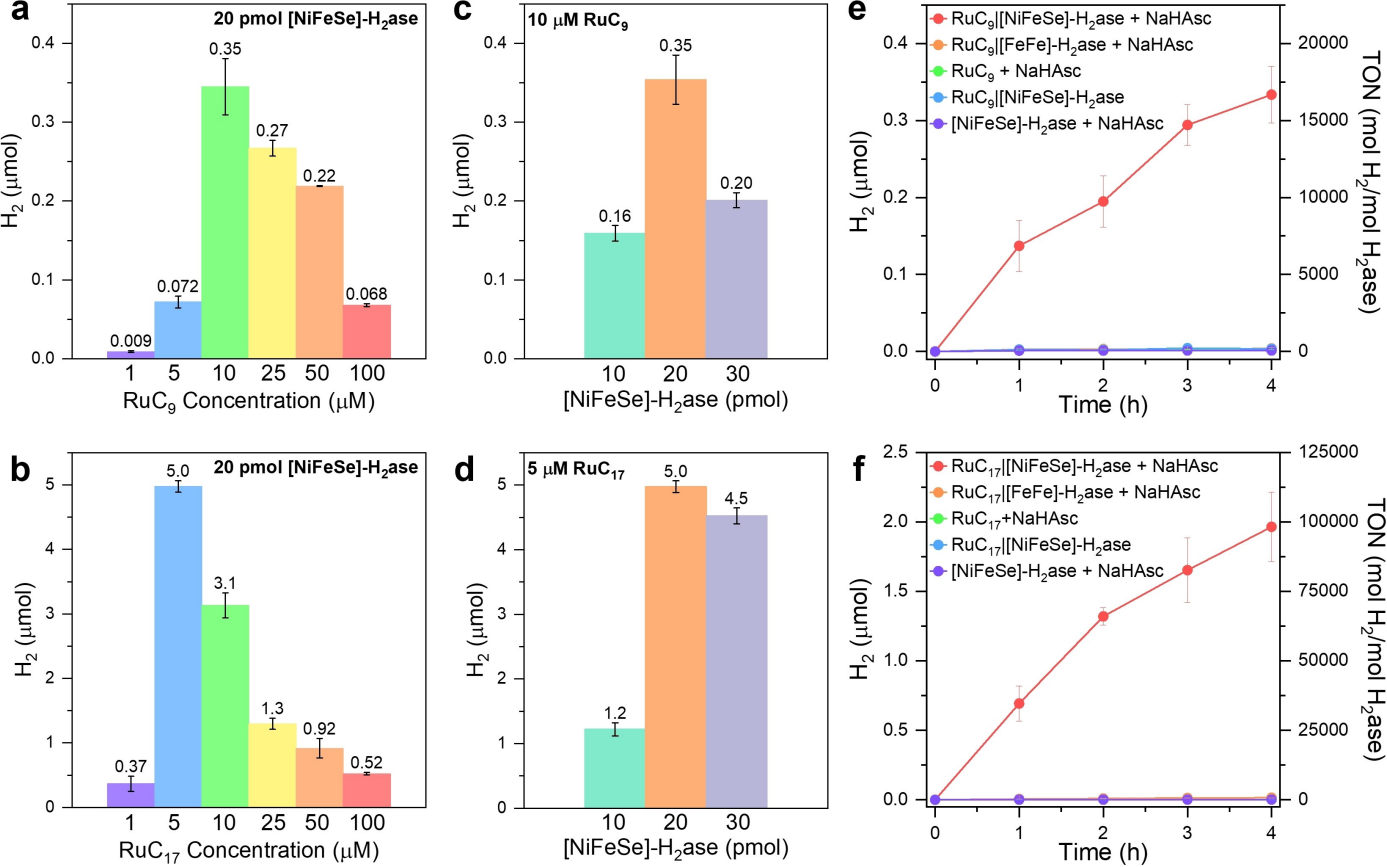
Photocatalytic H_2_ evolution for 24 hours using 20 pmol [NiFeSe]‐H_2_ase (40 nM) with (a) RuC_9_ and (b) RuC_17_ micelles employing surfactant concentrations ranging from 1 μM to 100 μM. Photocatalytic H_2_ evolution with RuC_*n*_|[NiFeSe]‐H_2_ase for 24 hours using (c) 10 μM RuC_9_ and (d) 5 μM RuC_17_ with different amounts of [NiFeSe]‐H_2_ase (10, 20, and 30 pmol). Time‐dependent photocatalytic H_2_ evolution of (e) 10 μM RuC_9_ and (f) 5 μM RuC_17_ with various control experiments. Conditions: anaerobic solution (0.5 mL) containing 0.1 M MOPS, 0.1 M NaHAsc, 20 pmol H_2_ase (either [NiFeSe] or [FeFe]), pH 7, AM 1.5G irradiation, 600 rpm stirring, 25 °C. Error bars represent the standard deviation for a sample size of 3.

The photocatalytic activity (amount of H_2_ produced over 24 hours) as a function of the surfactant concentration at a constant [NiFeSe]‐H_2_ase loading of 20 pmol (40 nM) shows a better catalytic activity of RuC_17_ than the other Ru surfactants when incorporated with H_2_ase (Figure 2a, b). The H_2_ yields display a maximum with respect to the surfactant concentration, with RuC_9_ showing a concentration‐dependent relationship, with a maximum of 350±36 nmol H_2_ at 10 μM RuC_9_ (Figure 2a). A comparable trend is observed for RuC_17_ with a maximum yield of 5.0±0.09 μmol H_2_ at 5 μM RuC_17_ (Figure 2b). The photocatalytic activity of RuC_17_ is an order of magnitude higher than RuC_9_ and corresponds to an enzyme‐TON of 250,000 for RuC_17_ after 24 hours. This value is three orders of magnitude higher than previously reported TONs for synthetic H_2_ase mimics with amphiphilic photosensitizers.[^14^, ^15^, ^16^]

The optimization of H_2_ yield as a function of [NiFeSe]‐H_2_ase loading was obtained at the optimal Ru‐surfactant concentration (Figure 2c, d). A loading of 20 pmol of [NiFeSe]‐H_2_ase was identified as optimal for both RuC_9_ and RuC_17_, presumably at a 1 : 1 micelle to enzyme ratio based on the micelle aggregation number. Time‐dependent photocatalytic H_2_ production was investigated to study the photostability of the system and control experiments performed to assess the significance of each component in the system (Figure 2e, f, Figure S10). The results show that all components are required, including RuC_*n*_ micelles, [NiFeSe]‐H_2_ase, and NaHAsc.

The fully assembled RuC_*n*_|[NiFeSe]‐H_2_ase systems produced 0.33±0.037 μmol of H_2_ (TON 16,500, based on H_2_ase) with RuC_9_ and 1.96±0.250 μmol of H_2_ (TON 98,000) with RuC_17_ after 4 hours under AM 1.5G irradiation at 25 °C (Figure 2e, f, Tables S1–S3).[^14^, ^15^, ^16^]

The 98,000 TON for H_2_ under 4 hours of irradiation corresponds to an overall quantum yield of 3.1 %, close to the recently developed artificial spherical chromatophore micellar system.^[26]^ During the initial 4 hours of a 24 hour experiment, RuC_9_ generated 94 % of the total amount of H_2_ produced, whereas RuC_17_ produced only 39 %, indicating that RuC_9_ has also a lower photostability than RuC_17_. The photostability of photocatalysts is a critical parameter for sustainable solar fuel production. While supramolecular structures are inherently dynamic, they are not fundamentally unstable or significantly affected by light irradiation, as evidenced by previous dynamic light scattering (DLS) studies.^[24]^ Instead, the observed decline in photocatalytic performance over time is more likely due to the chemical stability of the Ru‐based light absorber.^[43]^ No photo‐H_2_ production was observed after 24 hours for both systems (Figure S10a, d).[^24^, ^29^]

Control experiments using [FeFe]‐H_2_ase, an enzyme highly active for H_2_ production,^[44]^ instead of [NiFeSe]‐H_2_ase were conducted to investigate the electrostatic interaction between micelles and enzymes. The distal FeS cluster in [FeFe]‐H_2_ase is surrounded by a positively charged region containing arginine and lysine residues, which interact with the negatively charged region in ferredoxin for electron transfer in vivo.[^45^, ^46^] Both RuC_*n*_|[FeFe]‐H_2_ase systems show negligible amounts of H_2_ being produced (Figure 2e, f), supporting the proposed attractive electrostatic interaction between [NiFeSe]‐H_2_ase and micelles enabling interfacial DET.

A control experiment for photocatalytic H_2_ production using RuC_0_ (5 μM to 1 mM), a positively charged homogeneous photosensitizer without amphiphilicity, resulted only in marginal H_2_ production (<50 nmol H_2_ after 24 hours, Figure S11). This experiment confirms the beneficial role of the micelles as RuC_0_ does not effectively activate [NiFeSe]‐H_2_ase (20 pmol) and is consistent with previous findings of inefficient DET between homogeneous solutions of RuC_0_ and H_2_ase.[^29^, ^30^]

### Charge Carrier Dynamics in Micelle‐H_2_ase System

The reasons for the differences in performance between RuC_9_ and RuC_17_ micelles were investigated by nanosecond time‐resolved absorption and emission spectroscopy. Charge carrier dynamics were elucidated to gain insights into each reaction step and performance bottleneck of the photocatalytic system, at the concentrations of Ru surfactant that resulted in optimal photocatalytic performance (ca. 10 μM).

**Figure 3:**
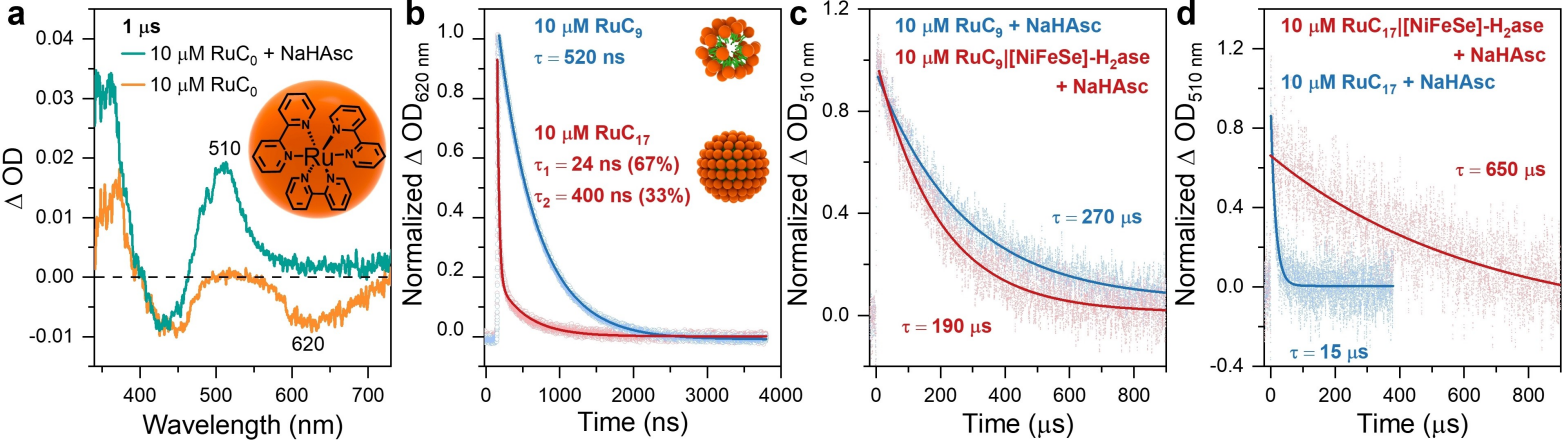
(a) UV–vis transient absorption spectra of RuC_0_ (10 μM) in the presence and absence of NaHAsc (0.1 M) at 1 μs obtained following 460 nm laser excitation (~10 ns, 7 mJ pulse^−1^) in Ar‐saturated aqueous MOPS (0.1 M) solution. (b) Normalized photoluminescence lifetimes at 620 nm of RuC_9_ (10 μM) and RuC_17_ (10 μM) following 460 nm laser excitation (~10 ns, 7 mJ pulse^−1^) in Ar‐saturated aqueous MOPS solution (0.1 M). Normalized kinetic traces for the reductively quenched (c) RuC_9_ and (d) RuC_17_ at 510 nm obtained in the presence of NaHAsc (0.1 M), with and without [NiFeSe]‐H_2_ase. Corresponding log‐linear plots are shown in Figure S16.

We studied first the electron transfer reactions between photosensitizer and sacrificial electron donor using time‐resolved spectroscopy (Figure S12a). Upon laser excitation at 460 nm of RuC_0_, the ground state bleach appears at 450 nm, along with the excited state absorption with a maximum at 370 nm and a long‐lived luminescence with an emission maximum at 620 nm.[^47^, ^48^] With the addition of NaHAsc (0.1 M), the primary diffusion‐controlled reductive quenching of photoexcited [Ru(bpy)_3_]^2+^* can be readily followed by the well‐known transient absorption features of [RuC_0_]^+^, including bleaching at 450 nm and absorption at 510 nm and 370 nm (Figure S12b). The comparison of these two transient absorption spectra at 1 μs is shown in Figure 3a. The corresponding emission lifetime at 620 nm with and without NaHAsc (0.1 M) changes from 600 ns to 160 ns (Figure S13a), suggesting a reductive quenching constant of 4.6×10^7^ M^−1^ s^−1^. The formation trace at 510 nm in the presence of NaHAsc (Figure S13b) also suggests direct electron transfer between photosensitizer and NaHAsc.

In the absence of NaHAsc, the emission lifetime behaviors of the amphiphilic photosensitizers RuC_9_ and RuC_17_ are significantly different (Figure 3b). Upon laser excitation at 460 nm, for 10 μM RuC_9_ in Ar‐saturated MOPS solution (0.1 M), the photoluminescence traces at 620 nm were fitted with a single‐exponential decay with time constant 520 ns, indicating that all of the excited states of RuC_9_ are in the similar situation as RuC_0_ (600 ns, Figures S13a and S14). This is a sign that the RuC_9_ micellar structure is poorly self‐assembled at the concentrations giving optimal H_2_ production. The photoluminescence traces of RuC_17_ at 620 nm were describable with a biexponential decay: two‐time constants obtained from fitting the data, 24 ns (67 %) and 400 ns (33 %). The biexponential decays can be explained in terms of self‐quenching between excited and ground states of RuC_17_,[^24^, ^49^] suggesting that there are two states of the RuC_17_ surfactants: a well self‐assembled micelles structure (67 %, 24 ns) and an unassembled state (33 %, 400 ns). From these photoluminescence results, we conclude that RuC_17_ has a much better self‐assembly ability than RuC_9_, resulting in a different optimal ratio and less stability for RuC_9_ compared to RuC_17_ in photocatalysis.

We finally employed time‐resolved spectroscopy to elucidate the electron transfer dynamics between the reduced photosensitizer and [NiFeSe]‐H_2_ase. Upon excitation of the metal to ligand charge transfer state of ruthenium, [RuC_9_*]^2+^ accepts an electron from NaHAsc, resulting in RuC_9_(I) and oxidized ascorbyl radical with a recombination lifetime of approximately 270 μs (Figure 3c, fitting residuals in Figure S15a). Furthermore, the addition of [NiFeSe]‐H_2_ase facilitates the decay of [RuC_9_]^+^ species, when 80 pmol [NiFeSe]‐H_2_ase was introduced into the 2 mL solution (under the same optimal photocatalytic condition), a kinetic trace at 510 nm of [RuC_9_]^+^ was observed to have a faster decay with time constant of approximately 190 μs, indicating electron transfer from [RuC_9_]^+^ to [NiFeSe]‐H_2_ase with diffusional control time constant 3.4×10^4^ M^−1^ s^−1^. (Figure 3c, fitting residuals in Figure S15b). For RuC_17_ in the absence of [NiFeSe]‐H_2_ase, due to the well self‐assembled structure, the charge recombination time between the reduced photosensitizer RuC_17_(I) and oxidized ascorbyl radical was much faster than that in RuC_9_, with time constant at around 15 μs (Figure 3d). When the same amount of [NiFeSe]‐H_2_ase was introduced into the solution, a kinetic trace at 510 nm of [RuC_17_]^+^ was observed to have a much slower decay with a time constant of approximately 650 μs, which was unexpected compared with the observed shorter time constant in RuC_9_. The reason for this behavior is likely that the addition of [NiFeSe]‐H_2_ase has a strong interaction with RuC_17_ and changes the structure of the well self‐assembled RuC_17_ micelle, leading to different charge recombination rates with and without [NiFeSe]‐H_2_ase. A similarly strong association between lipids and peptides has been recently reported, resulting in changes to membrane structure and properties.^[50]^ This suggests that the association between [NiFeSe]‐H_2_ase and RuC_17_ micelles is beneficial for the photocatalytic reaction.

### Photocatalytic CO_2_ Reduction with Micelle‐FDH Assembly

**Figure 4:**
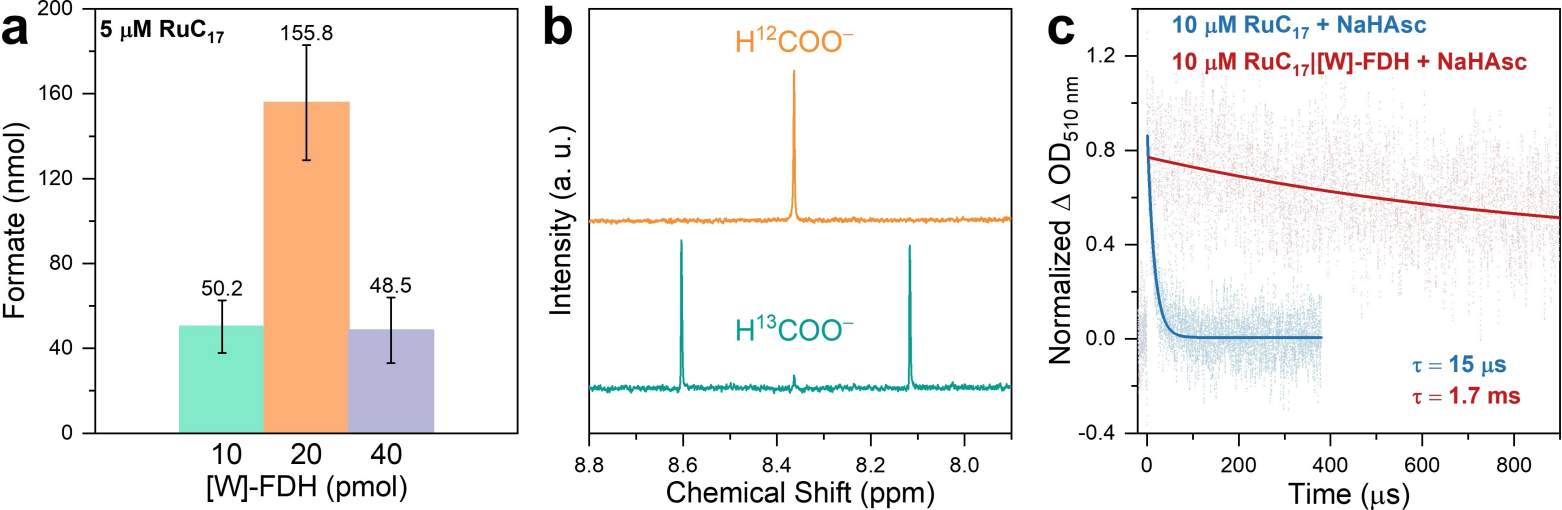
(a) Photocatalytic CO_2_ reduction to formate after 24 hours using RuC_17_ surfactants (5 μM) and different amounts of FDH (10, 20, and 40 pmol). (b) ^1^H NMR spectra (3 : 1 H_2_O/D_2_O, 400 MHz) of the solution after 24 hours photocatalysis using ^12^CO_2_/NaH^12^CO_3_ (orange line) and ^13^CO_2_/NaH^13^CO_3_ (green line). (c) Normalized kinetic traces for the reductively quenched RuC_17_ at 510 nm obtained in the presence of NaHAsc (0.1 M), with and without FDH. Corresponding log‐linear plot is shown in Figure S17. Conditions: anaerobic buffer solution (0.5 mL) containing CO_2_‐saturated NaHCO_3_ (0.1 M), NaHAsc (0.1 M), AM 1.5G irradiation, 600 rpm stirring, 25 °C. Error bars represent the standard deviation for a sample size of 3.

We next studied the integration of RuC_17_ micelles with [W]‐FDH for light‐driven formate production. Similar to [NiFeSe]‐H_2_ase, the distal FeS cluster in [W]‐FDH is surrounded by a negatively charged surface region comprising aspartic and glutamic acid residues,^[34]^ allowing for favorable interactions with the positively charged RuC_17_ micelles. Based on the optimization of RuC_17_ concentration above, we employed 5 μM RuC_17_ for micelle formation and optimized photocatalysis for 24 hours with varying amounts of FDH to 10, 20, and 40 pmol, yielding formate of 50.2, 155.8, and 48.5 nmol, respectively (Figure 4a). No by‐product such as H_2_, CO, or CH_4_ were detected owing to the highly selective nature of enzymes. Under the optimal conditions, 20 pmol FDH produced a TON for formate production of approximately 8,000 as determined by proton nuclear magnetic resonance (^1^H NMR) spectroscopy, corresponding to an overall quantum yield of 0.04 %. The TON for FDH is approximately 30 times lower than when using H_2_ase, which may be explained as follows; firstly, the inherent lower catalytic rate of FDH compared to H_2_ase; and secondly, while *Dv*H [NiFeSe]‐H_2_ase is found membrane‐bound in vivo due to its lipophilic character,^[51]^ there is no evidence of lipid‐protein interactions for *Dv*H [W]‐FDH, suggesting a potentially better native interaction of H_2_ase with micelles than FDH; and finally, the slower electron transfer to FDH that leads to more charge recombination with ascorbate radicals.

To unambiguously confirm the origin of the carbon source, isotopic labeling experiments utilizing ^13^CO_2_‐saturated electrolytes containing ^13^C‐labeled sodium bicarbonate (${\mathrm{NaH}}^{13}{\mathrm{CO}}_{3}$ , 0.1 M) were conducted. After 24 hours of photocatalysis, the primary product determined by ^1^H NMR spectroscopy (Figure 4b) was ^13^C‐formate (H^13^COO^−^), and the observed doublet peak had a ^13^C‐coupled proton constant ^1^ *J*_CH_=195 Hz. In contrast, photocatalysis employing ^12^CO_2_ yielded a singlet peak at 8.46 ppm. These findings confirm that CO_2_ serves as the sole carbon source for the light‐driven formate production.

Insights into the interaction between RuC_17_ micelles and FDH were obtained through nanosecond time‐resolved absorption spectroscopy. The decay of the reductively quenched [RuC_17_]^+^ at 510 nm was monitored, and the kinetic trace reveals a significantly longer lifetime of 1.7 ms for the micelle‐FDH complex, compared to the 15 μs lifetime of the pristine RuC_17_ micelles (Figure 4c). This prolonged lifetime suggests a distinct mechanism, potentially involving the deformation of the original micellar structure by FDH, similar to what was observed for the micelle‐H_2_ase complex (Figure 3d). This lifetime also shows the slow rate of electron transfer/recombination rates between RuC_17_ and FDH. It is noteworthy that the lifetime of the micelle‐FDH complex exceeds that of the micelle‐H_2_ase complex, which can be attributed to the intrinsic differences in catalytic rate between H_2_ase and FDH.[^33^, ^34^]

### Electrophoretic Analysis

**Figure 5:**
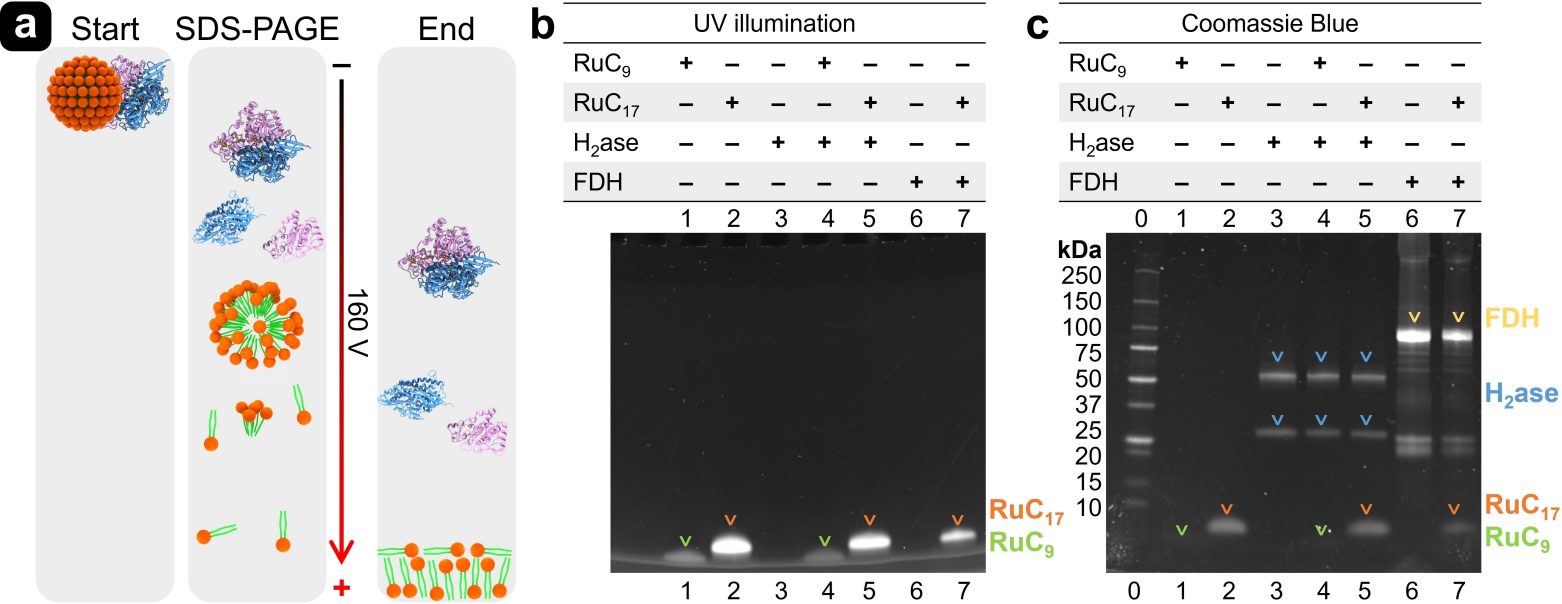
(a) Schematic illustration of SDS‐PAGE analysis of RuC_17_ micelle‐[NiFeSe]‐H_2_ase bio‐hybrids. SDS‐PAGE gel images of protein samples visualized by (b) UV illumination and (c) Coomassie Blue Stain. Lanes: 0. Precision Plus Protein™ Standards; 1. RuC_9_; 2. RuC_17_; 3. H_2_ase; 4. H_2_ase and RuC_9_; 5. H_2_ase and RuC_17_; 6. FDH; 7. FDH and RuC_17_. Raw SDS‐PAGE gel images can be found in Figures S18 and S19.

The micelle‐H_2_ase and micelle‐FDH assemblies have been studied by electrophoretic analysis (Figure 5a). In unstained SDS‐PAGE (sodium dodecyl sulfate‐polyacrylamide gel electrophoresis) gel under UV illumination, the fluorescence properties of RuC_0_ enabled the visualization of surfactants (Figure 5b). However, after electrophoresis, we observed that all band patterns were located at the bottom (low mass region) of the SDS‐PAGE gel (Figure 5b). RuC_9_ appears at a lower band than RuC_17_ due to the difference in molecular weight. This observation suggests that the structures of the electrostatically bonded micelle‐enzyme conjugates were disassembled into their surfactant molecules. This could be attributed to the presence of SDS and the electrical field applied during electrophoresis.

As an example, the disassembly process of a conjugated RuC_17_ micelle‐[NiFeSe]‐H_2_ase complex during SDS‐PAGE is illustrated in Figure 5a. It should be noted that the negatively charged SDS surfactant molecules associate with the surfaces of both enzymes and RuC_*n*_ surfactants, causing them to migrate towards the anode and separate based on their mass. Despite the limitations of SDS‐PAGE gel analysis resulting in disassembly of micelles and enzymes, the fluorescence emission of surfactants with different molecular weight, and the enzyme bands can be clearly observed after staining with Coomassie Blue (Figure 5c). The biohybrid assemblies were further characterized using Zeta potential measurements. The RuC_17_|H_2_ase and RuC_17_|FDH assemblies exhibited Zeta potentials of 8.5 mV and 11.2 mV, respectively, indicating the presence of electrostatic interactions. Similar behavior was observed in a polymer dot|[FeFe]H_2_ase biohybrid system.^[52]^

### Comparison of Related State‐of‐the‐Art Systems

**Figure 6:**
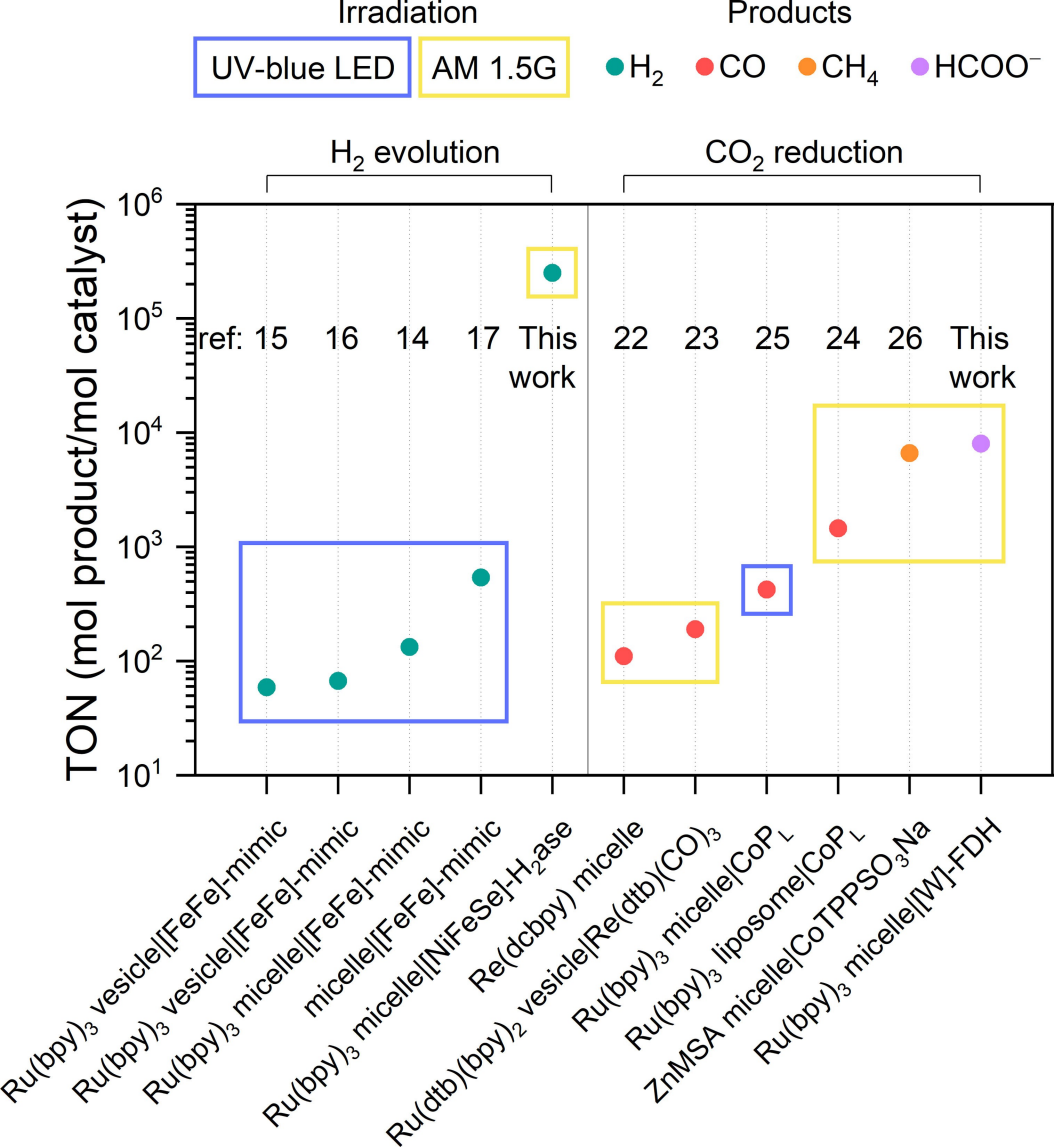
State‐of‐the‐art self‐assembled photocatalytic systems combing amphiphilic photosensitizers and co‐catalysts (Table S3).

This work presents the concept of biomimetic enzyme‐micelle self‐assembly for photocatalytic H_2_ production and CO_2_ reduction. Among supramolecular surfactant‐synthetic catalyst assemblies, the combination of efficient Ru‐based photosensitizers and selective enzymes leads to benchmark TONs for solar fuel synthesis using self‐assembled photocatalysts (Figure 6 and Table S3).

## Conclusions

We have demonstrated direct light activation of H_2_ase and FDH by employing self‐assembled micellar photosensitizers in vitro, an approach inspired by biological lipid‐protein interactions in vivo. The resulting biohybrids are functional thanks to strong interactions between the positively charged Ru‐dye‐micelles and the enzymes with a negative pocket around the electron entry site of the protein, resulting in direct solar H_2_ and formate production with benchmarking performance. We further investigated the influence of the alkyl chain length of the photosensitizer by comparing RuC_17_ with RuC_9_. We observed that RuC_17_ exhibited superior performance and stability when associated with enzymes, which can be attributed to its better self‐assembly and more positive surface charge compared to RuC_9_.

Mechanistic insights were obtained through nanosecond transient absorption and emission spectroscopy, which reveal the critical role of strong association in enabling DET between the positively charged RuC_17_ micelles and negatively charged H_2_ase and FDH enzymes. Overall, our findings highlight the potential of supramolecular surfactants as biomimetic scaffolds for biohybrid assembly to generate solar fuels or other photocatalytic reactions in the future.

## Supporting Information

Experimental section, supporting Figures and tables are provided in the Supporting Information.

## Conflict of Interests

The authors declare no conflict of interest.

1

## Supporting information

As a service to our authors and readers, this journal provides supporting information supplied by the authors. Such materials are peer reviewed and may be re‐organized for online delivery, but are not copy‐edited or typeset. Technical support issues arising from supporting information (other than missing files) should be addressed to the authors.

Supporting Information

## Data Availability

The raw data supporting the findings of this study can be accessed through the University of Cambridge data repository: https://doi.org/10.17863/CAM.115951.
