# Supplementary material for: Bio‐Inspired Self‐Assembly of Enzyme‐Micelle Systems for Semi‐Artificial Photosynthesis
Source: Angew Chem Int Ed Engl. 2025 Mar 3;64(18):e202424222. doi: 10.1002/anie.202424222 (PMC12036810; doi:10.1002/anie.202424222)
Supplement: Supplementary file 1 — Supporting Information [file ANIE-64-e202424222-s001.pdf]

Angewandte Chemie
Eine Zeitschrift der Gesellschaft Deutscher Chemiker

Supporting Information

Bio-Inspired Self-Assembly of Enzyme-Micelle Systems for Semi-Artificial Photosynthesis

*Y. Liu, S. Rodríguez-Jiménez, H. Song, A. Pannwitz, D. Kim, A. M. Coito, R. R. Manuel, S. Webb, L. Su, S. A. Bonke, R. D. Milton, I. A. C. Pereira, S. Bonnet, L. Hammarström, E. Reisner**

Supporting Information

Angewandte International Edition Chemie
www.angewandte.org

WILEY-VCH

Supporting Information for

Bio-Inspired Self-Assembly of Enzyme-Micelle Systems for Semi-Artificial Photosynthesis

Yongpeng Liu,[a] Santiago Rodríguez-Jiménez,[a] Hongwei Song,[b] Andrea Pannwitz,[c] Dongseok Kim,[a] Ana M. Coito,[d] Rita R. Manuel,[d] Sophie Webb,[e,f] Lin Su,[a] Shannon A. Bonke,[a] Ross D. Milton,[e,f] Inês A. C. Pereira,[d] Sylvestre Bonnet,[c] Leif Hammarström,[b] and Erwin Reisner*[a]

[a] Dr. Y. Liu,+ Dr. S. Rodríguez-Jiménez,+ Mr. D. Kim, Dr. L. Su, Dr. S. A. Bonke, Prof. E. Reisner
Yusuf Hamied Department of Chemistry,
University of Cambridge,
Lensfield Road, Cambridge CB2 1EW, UK
E-mail: reisner@ch.cam.ac.uk

[b] Dr. H. Song,+ Prof. L. Hammarström
Department of Chemistry − Angstrom Laboratory,
Uppsala University,
751 20 Uppsala, Sweden

[c] Dr. A. Pannwitz, Prof. S. Bonnet
Leiden Institute of Chemistry,
Leiden University,
2333 CC Leiden, The Netherlands

[d] Ms. A. M. Coito, Dr. R. R. Manuel, Prof. I. A. C. Pereira
Instituto de Tecnologia Química e Biológica António Xavier (ITQB NOVA),
Universidade NOVA de Lisboa,
2780-157 Oeiras, Portugal

[e] Ms. S. Webb, Prof. R. D. Milton
Department of Inorganic and Analytical Chemistry,
University of Geneva,
1211 Geneva 4, Switzerland

[f] Ms. S. Webb, Prof. R. D. Milton
National Centre of Competence in Research (NCCR) Catalysis,
University of Geneva,
1211 Geneva 4, Switzerland

[+] These authors contributed equally to this work.

WILEY-VCH

How to cite: *Angew. Chem. Int. Ed.* **2025**, e202424222

https://doi.org/10.1002/anie.202424222

Contents

S1 Experimental Section S6

S1.1 Materials S6

S1.2 Isolation and Purification of Enzymes S7

S1.3 Physical Characterization S7

S1.4 Photocatalysis and Product Quantification S7

S1.5 Quantum Yield Calculation S8

S1.6 Isotopic Labeling S9

S1.7 Nanosecond Transient Spectroscopy S9

S1.8 Electrophoresis S10

S1.9 Synthetic Methods and Characterizations S10

S2 NMR Spectra of Prepared Ligand and Photosensitizers S14

S3 UV−vis Absorption Spectra of Photosensitizers S19

S4 Estimation of Micelle Aggregation Numbers S20

S5 Representative Steady-State Fluorescence Spectra S21

S6 Photocatalytic H_2 Production with RuC_9/RuC_{17} and [NiFeSe]-H_2ase S22

S7 Table of Photocatalytic H_2 Evolution using RuC_9. . . . . S23

S8 Table of Photocatalytic H_2 Evolution using RuC_{17} S23

S9 Comparison among State-of-the-art Self-Assembled Photocatalytic Systems . S24

S10 Photocatalytic H_2 Production with RuC_0 and [NiFeSe]-H_2ase. S25

S11 Additional Transient Absorption and Emission S26

S12 Raw SDS-PAGE Gel Images. S30

References S32

List of Figures

S1 Synthetic procedure to alkylated ruthenium bipyridine photosensitiser RuC_9 . . . . . . S11

S2 Synthetic procedure to bpy_{C17} and alkylated ruthenium bipyridine photosensitiser RuC_{17} S13

S3 ^1H NMR spectrum of bpy_{17} ligand in $CDCl_3$ S14

S4 ^1H NMR spectrum of RuC_9 in D_2O S15

S5 ^1H NMR spectrum of RuC_9 in CD_3OD S16

S6 ^1H NMR spectrum of RuC_{17} in D_2O S17

S7 ^1H NMR spectrum of RuC_{17} in CD_3OD S18

S8 UV−vis absorption spectra of photosensitizers RuC_0, RuC_9 and RuC_{17} in water S19

S9 Representative steady-state fluorescence spectra for (a) RuC_9 and (b) RuC_{17} S21

S10 Time-dependent photocatalytic H_2 evolution of 10 μM RuC_9 and 5 μM RuC_{17} with various control experiments for 25 hours S22

S11 Time-dependent photocatalytic H_2 evolution with different concentrations of RuC_0 and 20 pmol H_2ase S25

S12 UV−vis transient absorption spectra of (a) 10 μM RuC_0 and (b) 10 μM RuC_0 + 0.1 M NaHAsc obtained following 460 nm laser excitation. . . . S26

S13 (a) Normalized time-resolved emission of 10 μM RuC_0 with and without 0.1 M NaHAsc. (b) The RuC_0 formation kinetic trace at 510 nm in the presence of 0.1 M NaHAsc. Condition: excitation wavelength at 460 nm (10 ns, 7 mJ per pulse) in Ar-saturated 0.1 M MOPS buffer. . . . S26

S14 UV–vis transient absorption spectra of RuC_9 (10 μM) in the presence of NaHAsc (0.1 M). S27

S15 The corresponding fitting residuals for **Figure 3c**. S28

S16 Corresponding Log plots for **Figure 3b−d**. S28

S17 Corresponding Log plot for **Figure 4c**. S29

S18 Raw SDS-PAGE gel image for **Figure 5b** S30

S19 Raw SDS-PAGE gel image for **Figure 5c** S31

List of Tables

S1 Table of photocatalytic H_2 evolution using RuC_9 . . . . S23

S2 Table of photocatalytic H_2 evolution using RuC_{17} S23

S3 Comparison among state-of-the-art self-assembled photocatalytic systems combing amphiphilic photosensitizers and co-catalysts for H_2 evolution and CO_2 reduction S24

S1 Experimental Section

S1.1 Materials

The chemicals and materials were purchased from commercial suppliers and used without further purification: CO_2 and N_2 gas bottles (2% CH_4 as internal standard, BOC), carbon-^{13}C dioxide ($^{13}CO_2$, Sigma Aldrich, 99.0 atom % ^{13}C), 3-(N-morpholino)propanesulfonic acid (MOPS, $C_7H_{15}NO_4S$, Sigma Aldrich, ≥99.5%), MOPS sodium salt ($C_7H_{14}NNaO_4S$, Sigma Aldrich, ≥99.5%), (+)-sodium L-ascorbate (NaHAsc, $C_6H_7NaO_6$, Sigma Aldrich, ≥99.0%), sodium bicarbonate ($NaHCO_3$, Sigma Aldrich, ≥99.9%), sodium bicarbonate-^{13}C ($NaH^{13}CO_3$, Sigma Aldrich, 98 atom % ^{13}C), 4,4'-dimethyl-2,2'-dipyridyl ($C_{12}H_{12}N_2$, Thermo Scientific Chemicals, 98%), 1-bromohexadecane ($C_{16}H_{33}Br$, AK Scientific, 97%), tris(2,2'-bipyridyl)ruthenium(II) dichloride hexahydrate, (RuC_0, $C_{30}H_{24}Cl_2N_6Ru \cdot 6H_2O$, Sigma Aldrich, 99.95% trace metals basis), cis-dichlorobis(2,2'-bipyridine)ruthenium(II) dihydrate ($C_{20}H_{20}Cl_2N_4O_2Ru$, Alfa Aesar, ≥19% Ru), 4,4'-dinonyl-2,2'-dipyridyl ($C_{28}H_{44}N_2$, Sigma Aldrich, 97%), lithium diisopropylamide ($C_6H_{14}LiN$, Acros Organics, 2 M in THF/n-heptane/ethylbenzene), DL-Dithiothreitol (DTT, $HSCH_2CH(OH)CH(OH)CH_2SH$, Fisher, ≥98.0%), potassium trioxalatoferrate(III) trihydrate ($K_3[Fe(C_2O_4)_3]{\cdot}3H_2O$, Thermo Scientific Chemicals, 99.7%), 1,10-phenanthroline ($C_{12}H_8N_2$, Alfa Aesar, 99%), sulfuric acid solution (H_2SO_4, Honeywell Fluka, for HPLC 49-51%), and rubber septa (Subaseal). MilliQ H_2O (18.2 MΩ cm) was used for all the experiments. All synthetic procedures involving air- or moisture-sensitive materials were executed within an inert N_2 atmosphere, employing the Schlenk techniques. The solvents employed in the experiments were either purchased in a dried state (e.g., DMF) or dried using conventional purification methods under an inert atmosphere.

S1.2 Isolation and Purification of Enzymes

[NiFeSe]-H_2ase and [W]-FDH from *Dv*H were expressed and purified according to a previously reported method.[S1,S2] [FeFe]-H_2ase from *Cp*I was produced as reported previously.[S3] All purification steps were performed under anoxic conditions.

S1.3 Physical Characterization

^{1}H and ^{13}C NMR spectra were collected with a Bruker 400 MHz NMR spectrometer at 25 °C. Chemical shifts for ^{1}H NMR spectra are referenced relative to residual protons in the deuterated aqueous solvent (δ = 4.79 ppm, Eurisotop). Elemental analyses were carried out using a Perkin-Elmer 240 Elemental Analyzer. High-resolution mass spectra were recorded using a Synapt G2-Si high-definition mass spectrometer. UV–vis spectra were collected using a Cary 60 UV–vis spectrometer. Attenuated total reflectance Fourier-transform infrared spectra were recorded on a Nicolet iS50 spectrometer. Fluorimetry was performed on an Edinburgh Instruments Spectrofluorometer FS5 equipped with a Visible PMT-900 detector. Zeta potentials were determined by using a Malvern Zetasizer Nano ZS spectrometer equipped with a red laser (632.8 nm).

S1.4 Photocatalysis and Product Quantification

In a photoreactor, RuCn surfactants were dissolved in an aqueous solution containing 0.1 M NaHAsc in either 0.1 M MOPS (for H_2ase) or 0.1 M $NaHCO_3$ (for FDH) for a total volume of 0.5 mL, after which H_2ase was added. For photocatalysis involving FDH, FDH (40 μM) was incubated with the same volume of DTT (50 mM in 50 mM MOPS, pH 7) for 20 min prior adding into the photoreactor, and the solution was purged with CO_2 for 10 min. All of the photoreactors were assembled and sealed in an anaerobic glovebox. During photocatalysis, the photoreactors were illuminated under simulated AM 1.5G irradiation and stirred at 600 rpm at 25 °C. Gaseous products were analyzed by headspace gas analysis

using a Shimadzu Tracera GC-2010 Plus with a barrier discharge ionization detector kept at 40 °C using helium carrier gas. Aliquots of 100 μL of the headspace gas were removed from the sealed photocatalytic vials using a gastight syringe (Hamilton) for GC analysis. The liquid product was analyzed by ^{1}H NMR spectroscopy. After 24 h of simulated AM 1.5G irradiation, 450 μL of the aqueous reaction solution was transferred into an NMR tube, together with 150 μL of D_2O containing 0.05% wt/v of 3-(trimethylsilyl)propionic-2,2,3,3-d_4 acid, sodium salt, as the internal standard. Subsequently, the ^{1}H NMR spectra were collected with a 400 MHz NMR spectrometer.

S1.5 Quantum Yield Calculation

The number of incident photons was measured using $K_3[Fe(C_2O_4)_3]$ as a chemical actinometer, following previously reported methods.[S4–S6] The calculation was based on known parameters for the Fe^{2+} complex, including a molar absorption coefficient of 11,100 M^{-1} cm^{-1} and a quantum yield of Fe^{2+} formation of 1.21.[S4–S6] The incident photon number was determined to be $(1.9 \pm 0.2) \times 10^{19}$ photons per hour. The overall quantum yield ($\phi_{overall}$) for proton reduction to H_2 is defined as:

$$\phi_{overall}(\%) = \frac{2 \times n(H_2)}{\text{Incident photons}} \times 100$$

On a Ru(bpy)$_3$ micelle|[NiFeSe]-H_2ase assembly, TONs of 98,000 (4 h) and 250,000 (24 h) resulting in a $\phi_{overall}$ of 3.11% and 1.32%, respectively.

For CO_2-to-formate conversion, $\phi_{overall}$ is defined as:

$$\phi_{overall}(\%) = \frac{2 \times n(\text{formate})}{\text{Incident photons}} \times 100$$

Taking the 24 hours TON of 8,000 for formate on a Ru(bpy)$_3$ micelle|FDH assembly, a $\phi_{overall}$ of 0.04% is observed.

S1.6 Isotopic Labeling

Photocatalysis experiments with 0.1 M $NaH^{13}CO_3$ and 0.1 M NaHAsc aqueous solution with $^{13}CO_2$ as the headspace gas were performed. After 24 h of simulated AM 1.5G irradiation, the solution was transferred to an NMR tube and a ^{1}H NMR spectrum was collected with a 400 MHz NMR spectrometer.

S1.7 Nanosecond Transient Spectroscopy

Nanosecond transient absorption and emission experiments were conducted using a setup consisting of the third harmonic output of a frequency-doubled Q-switched Nd:YAG (neodymium-doped yttrium aluminum garnet, Nd:$Y_3Al_5O_{12}$) laser with an optical parametric oscillator (OPO) to generate 460 nm excitation pulses. Time-resolved spectra and kinetic traces in the nano-to-microsecond range were acquired using an Ekspla NT340 system delivering 460 nm laser pulses at 7 mJ per pulse energy. The laser was coupled to an LP 920 detection system (Edinburgh Instruments) equipped with a pulsed XBO 450 W xenon Arc Lamp (Osram), providing white light for probing. Transient signals were detected using an iStar charge-coupled device (CCD) camera (Andor Technology) and an LP920-K photomultiplier (PMT) detector connected to a Tektronix TDS 3052 oscilloscope (500 MHz 5 GS/s). Transient absorption data were acquired using LP 900 software and processed with the Origin 2021b software. For spectral and trace averaging the sample was excited at ca. 0.5 Hz. For kinetic traces in the millisecond range, a Quantel Brilliant B laser with an Opotek OPO was utilized to generate 460 nm laser pulses with an energy of 7 mJ per pulse. The probe light, emitted from a constantly illuminated 150 W Xe lamp in a flash photolysis spectrometer (Applied Photophysics LKS.60), was single wavelength. To minimize sample excitation by the probe light, two monochromators were employed. The first monochromator was set to the desired detection wavelength prior to reaching the sample, while the second monochromator was positioned after the sample. The absorption difference of samples at specific wavelengths was monitored using a PMT Hamamatsu R928 detector and digitized with an Agilent Tech-

nologies Infinium digital oscilloscope (600 MHz). Transient absorption data acquisition was performed within the Applied Photophysics LKS software package. All transient absorption and emission measurements were carried out at room temperature using a gas-tight quartz cell cuvette with a 1.0 cm optical path length. The cuvette contained 2 mL solution, and the enzyme-micelle bio-hybrids were assembled inside an anaerobic glovebox. For measurements involving H_2ase, the solution consisted of 0.1 M MOPS saturated with Ar, while measurements involving FDH utilized a solution of 0.1 M $NaHCO_3$ saturated with CO_2.

S1.8 Electrophoresis

Enzymes and surfactants were prepared at the optimal photocatalytic conditions (5 μM RuC17 and 40 nM [NiFeSe]-H_2ase). Protein electrophoresis was performed as previously described. Briefly, 5-10 μL of protein samples were mixed with an equal volume of 2× Laemmli Sample Buffer, loaded onto an SDS-PAGE gel (4-20% Mini-PROTEAN TGX Precast Protein Gels) with a protein ladder (Unstained Protein Standards), and subjected to vertical electrophoresis (160 V, 45 min) using 1× Tris/Glycine/SDS as the running buffer. For UV illumination imaging, gels were washed three times with deionized water after electrophoresis. For Coomassie Blue staining, GelCode Blue Stain Reagent (Thermo Scientific) was used following the user guide. Briefly, the gel was immersed in the stain reagent for 1 hour after UV illumination imaging, then changed to deionized water overnight. Both UV illumination and Coomassie Blue images were captured using the ChemiDoc Imaging Systems (Bio-Rad). All chemicals and supplies were obtained from Bio-Rad unless otherwise indicated.

S1.9 Synthetic Methods and Characterizations

Synthesis of RuC$_9$. The synthesis was performed following reported protocol from ref,[S7] as shown in Figure S1, and the physicochemical characterization matches that reported in the same ref.

EA results: Calculated for $C_{48}H_{60}N_6Cl_2Ru{\cdot}2.45H_2O$ (M = 937.16 g mol^{-1}): C 61.52,

H 6.98, N 8.97 %. Found: C 61.47, H 6.89, N 8.91 %. **^{1}H NMR (D_2O, 400 MHz)**: δ (ppm) = 8.49-8.45 (t, 4H$_{d'}$), 8.09 (s, 2H$_d$), 7.99-7.91 (q, 4H$_{c'}$), 7.69-7.68 (d, 2H$_a$), 7.60-7.59 (d, 4H$_{a'}$), 7.33-7.25 (dt, 4H$_{b'}$), 7.15-7.13 (d, 2H$_b$), 2.62-2.59 (t, 4H$_e$, 2 × CH_2), 1.46-1.38 (p, 4H$_f$, 2 × CH_2), 1.08-0.88 (m, 24H$_g$, 12 × CH_2), 0.52-0.48 (t, 6H$_h$, 2 × CH_3). **^{1}H NMR (CD_3OD, 400 MHz)**: δ (ppm) = 8.71-8.69 (d, 4H$_{d'}$), 8.62-8.61 (d, 2H$_d$), 8.14-8.10 (tt, 4H$_{c'}$), 7.82-7.81 (m, 4H$_{a'}$), 7.64-7.62 (d, 2H$_a$), 7.50-7.47 (ddd, 4H$_{b'}$), 7.35-7.33 (dd, 2H$_b$), 2.87-2.83 (t, 4H$_e$, 2 × CH_2), 1.77-1.69 (p, 4H$_f$, 2 × CH_2), 1.44-1.25 (m, 24H$_g$, 12 × CH_2), 0.90-0.87 (t, 6H$_h$, 2 × CH_3). **ESI-MS (+, methanol)**: m/z calculated for $C_{48}H_{60}N_6Ru^{2+}$ (i.e. $[M^{2+} - 2 \times Cl^-]^{2+}$): 411.1956, found: 411.2055. **UV-vis (Milli-Q water)**: λ_{max} (nm) = 456, ε (M^{-1} cm^{-1}) = 14.0 × 10^3. **ATR-FTIR**: ν (cm^{-1}) = 3354 (broad), 3100, 3068, 3008, 2922, 2852, 1601, 1463, 1442, 1418, 1308, 1267, 1241, 1123, 1025, 770, 732.

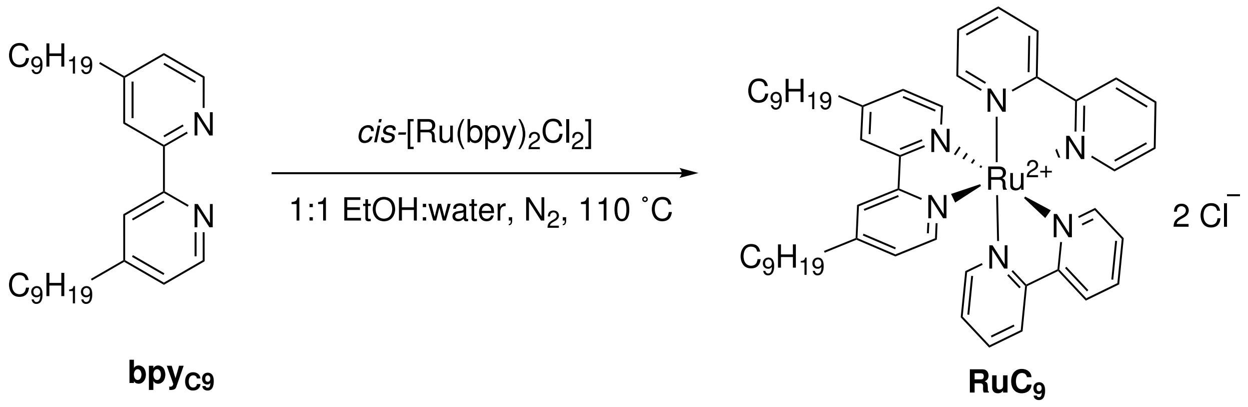

Figure S1: Synthetic procedure to alkylated ruthenium bipyridine photosensitiser RuC_9.

Synthesis of $\mathbf{bpy_{C17}}$. The synthesis was performed following reported protocol from ref,[S8] as shown in Figure S2.

EA results: Calculated for $C_{44}H_{76}N_2{\cdot}0.25H_2O$ (M = 637.60 g mol^{-1}): C 82.89, H 12.09, N 4.39 %. Found: C 82.80, H 12.26, N 4.46 %. **^{1}H NMR ($CDCl_3$, 400 MHz)**: δ (ppm) = 8.56-8.55 (d, 2H$_a$), 8.23 (s, 2H$_c$), 7.14-7.12 (dd, 2H$_b$), 2.71-2.67 (t, 4H$_d$, 2 × CH_2), 1.72-1.65 (m, 4H$_e$, 2 × CH_2), 1.37-1.20 (m, 56H$_f$, 28 × CH_2), 0.89-0.86 (t, 6H$_g$, 2 × CH_3). **ESI-MS (+, methanol)**: m/z calculated. for $C_{44}H_{77}N_2^+$ (i.e. $[M + H^+]^+$) 633.6087, found: 633.6108. **UV-vis ($CHCl_3$)**: λ_{max} (nm) = 284. **ATR-FTIR**: ν (cm^{-1}) = 3060, 2954, 2914, 2846, 1597, 1546, 1469, 1420, 1384, 1111, 993, 896, 827, 719, 668, 591, 508.

Synthesis of $\mathbf{RuC_{17}}$. The synthesis was carried out following reported protocol from ref,[S8] as shown in Figure S2, and the physicochemical characterization matches that reported in the same ref.

EA results: Calculated for $C_{48}H_{60}N_6Cl_2Ru{\cdot}7H_2O$ (M = 1243.56 g mol^{-1}): C 61.81, H 8.59, N 6.76 %. Found: C 61.67, H 8.29, N 6.50 %. **^{1}H NMR (D_2O, 400 MHz)**: δ (ppm) = 8.42 (s, 4H$_{d'}$), 8.11 (s, 2H$_d$), 7.90-7.83 (d, 4H$_{c'}$), 7.63-7.58 (d + d, 2H$_a$ + 4H$_{a'}$), 7.27 (s, 4H$_{b'}$), 7.05 (s, 2H$_b$), 2.52 (s, 4H$_e$), 1.39 (s, 4H$_f$), 1.13 (m, 56H$_g$, 28 × CH_2), 0.72 (s, 6H$_h$, 2 × CH_3). **^{1}H NMR (CD_3OD, 400 MHz)**: δ (ppm) = 8.72-8.70 (dd, 4H$_{d'}$), 8.63 (dd, 2H$_d$), 8.14-8.10 (tt, 4H$_{c'}$), 7.83-7.81 (dt, 4H$_{a'}$), 7.84-7.83 (d, 2H$_a$), 7.51-7.46 (ddd, 4H$_{b'}$), 7.35-7.33 (dd, 2H$_b$), 2.87-2.83 (t, 4H$_e$, 2 × CH_2), 1.76-1.70 (p, 4H$_f$, 2 × CH_2), 1.45-1.21 (m, 56H$_g$, 28 × CH_2), 0.91-0.88 (t, 6H$_h$, 2 × CH_3). **ESI-MS (+, methanol)**: m/z calculated for $C_{64}H_{92}N_6Ru^{2+}$ (i.e. $[M^{2+} - 2 \times Cl^-]^{2+}$): 523.3208, found: 523.3248. **UV-vis (methanol)**: λ_{max} (nm) = 454, ε (M^{-1} cm^{-1}) = 13.5 × 10^3. **UV-vis (Milli-Q water)**: λ_{max} (nm) = 458, ε (M^{-1} cm^{-1}) = 14.0 × 10^3. **ATR-FTIR**: ν (cm^{-1}) = 3379 (broad), 3070, 2918, 2850, 1616, 1611, 1464, 1444, 1422, 1313, 1243, 1161, 1123, 1026, 774, 731.

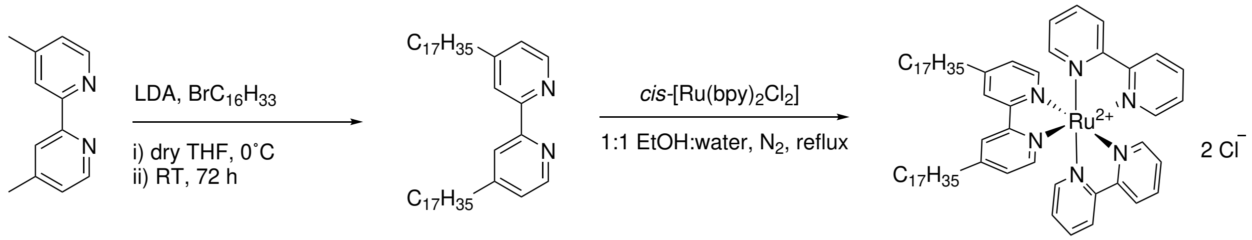

bpy$_{C1}$ **bpy$_{C17}$** **RuC$_{17}$**

Figure S2: Synthetic procedure to bpy$_{C17}$ and alkylated ruthenium bipyridine photosensitiser RuC$_{17}$.

S2 NMR Spectra of Prepared Ligand and Photosensitizers

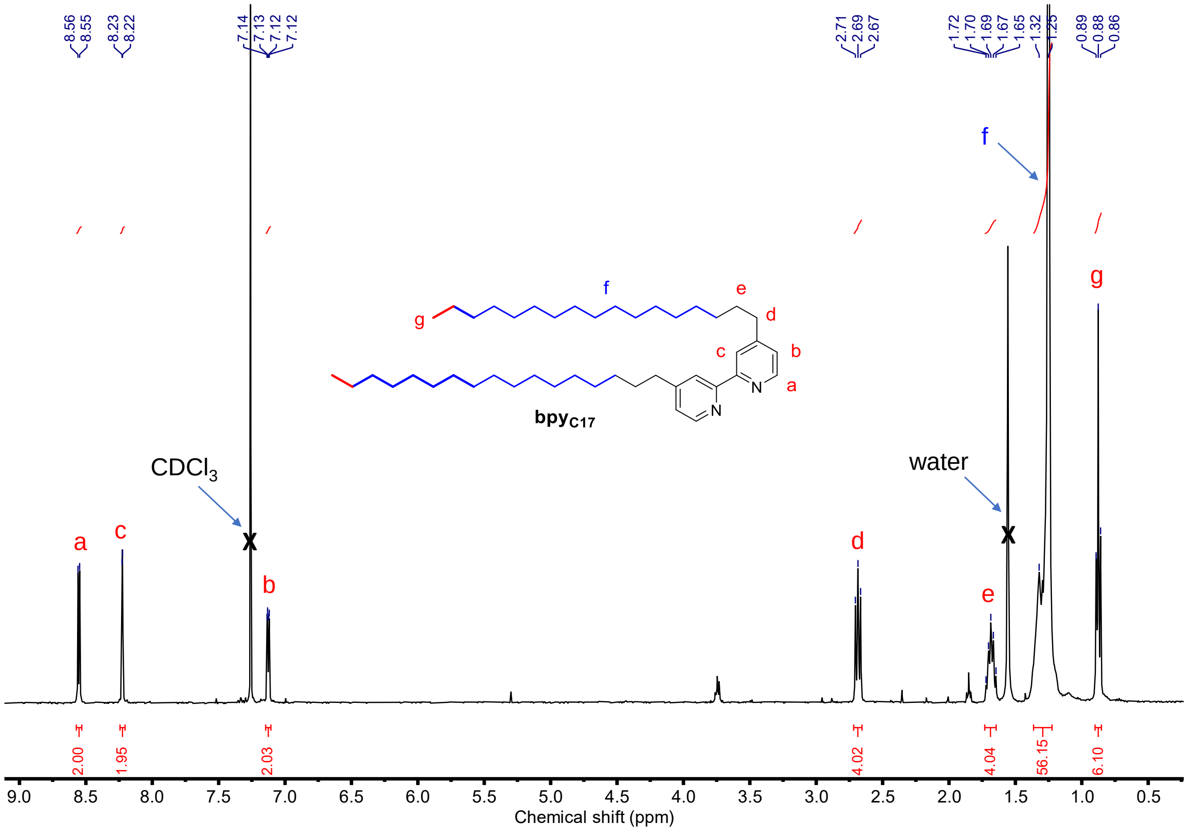

Figure S3: ^{1}H NMR spectrum of bpy$_{17}$ ligand in $CDCl_3$.

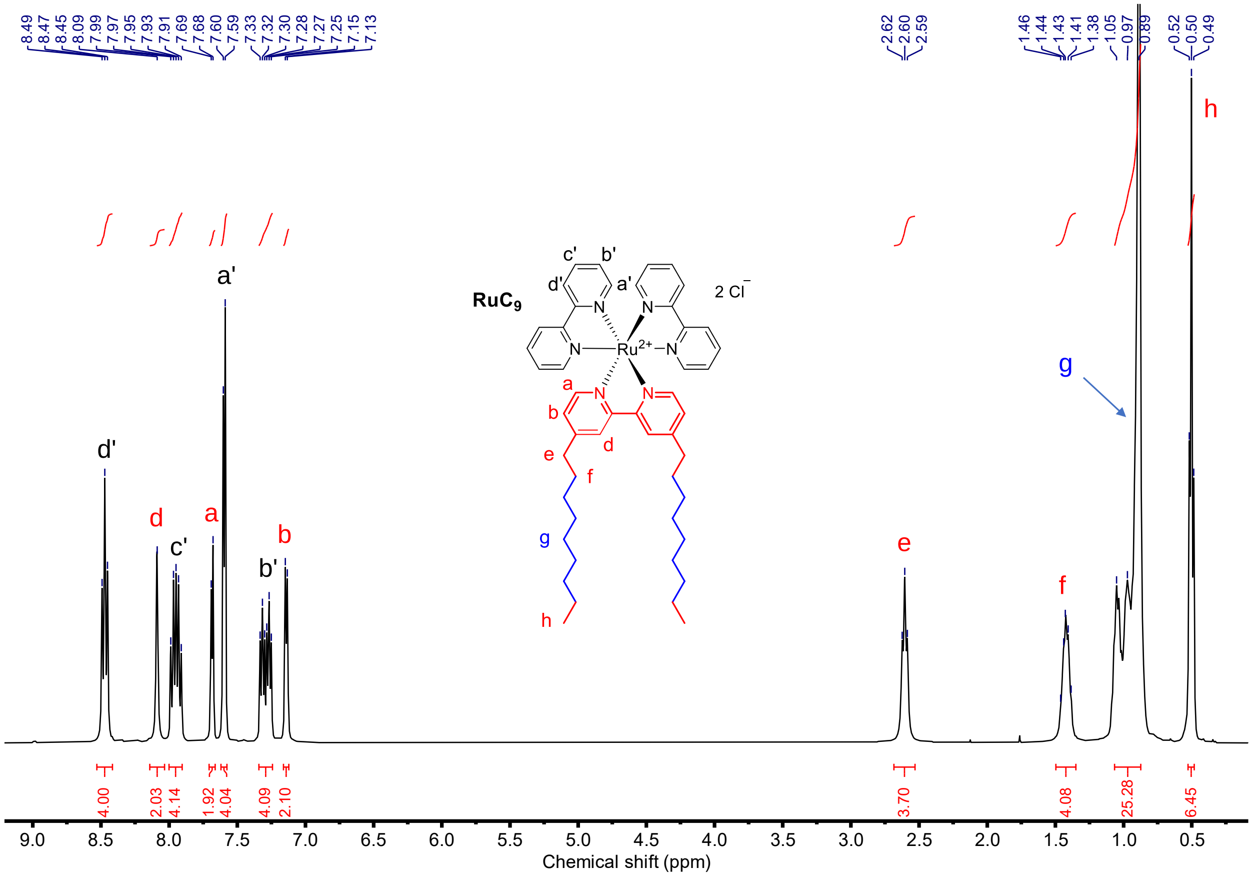

Figure S4: ^{1}H NMR spectrum of RuC_9 in D_2O.

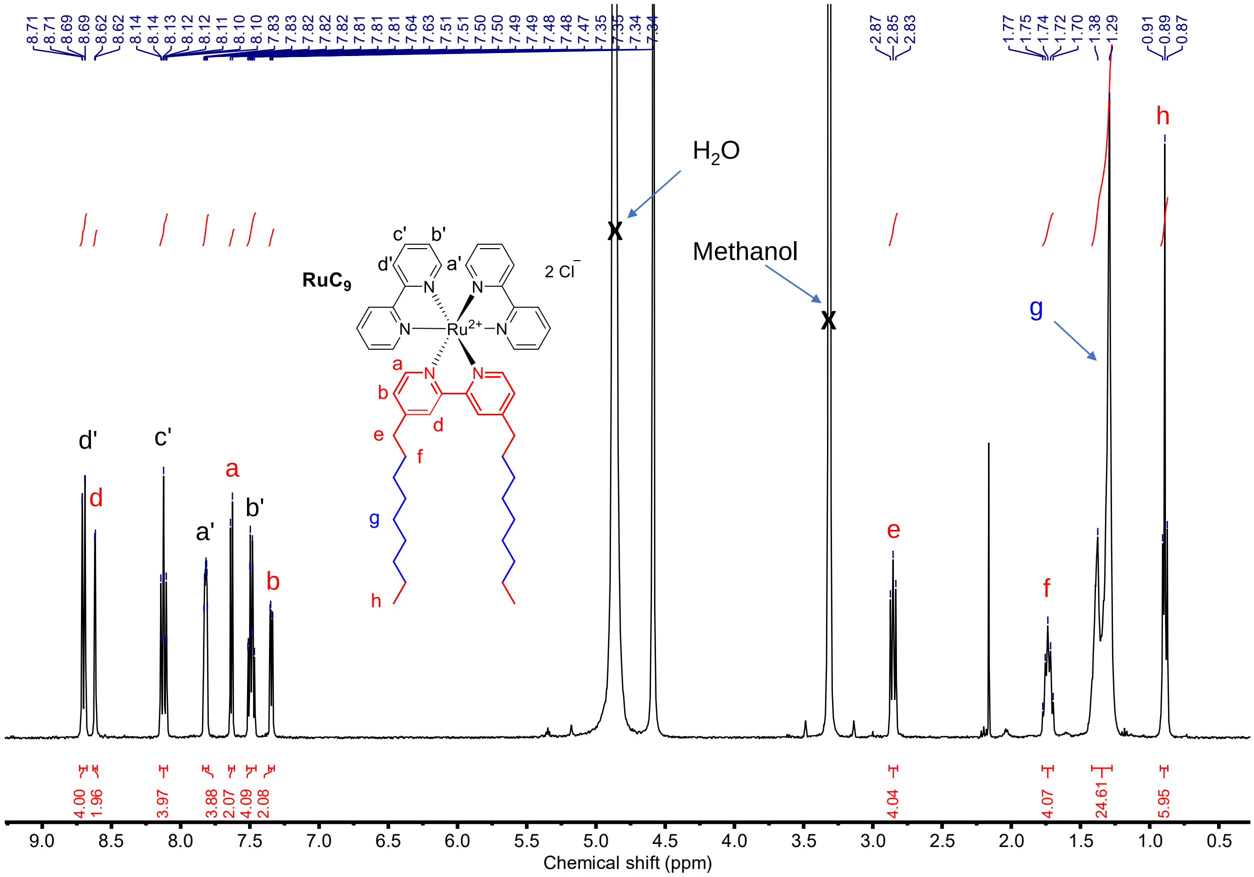

Figure S5: ^{1}H NMR spectrum of RuC_9 in CD_3OD.

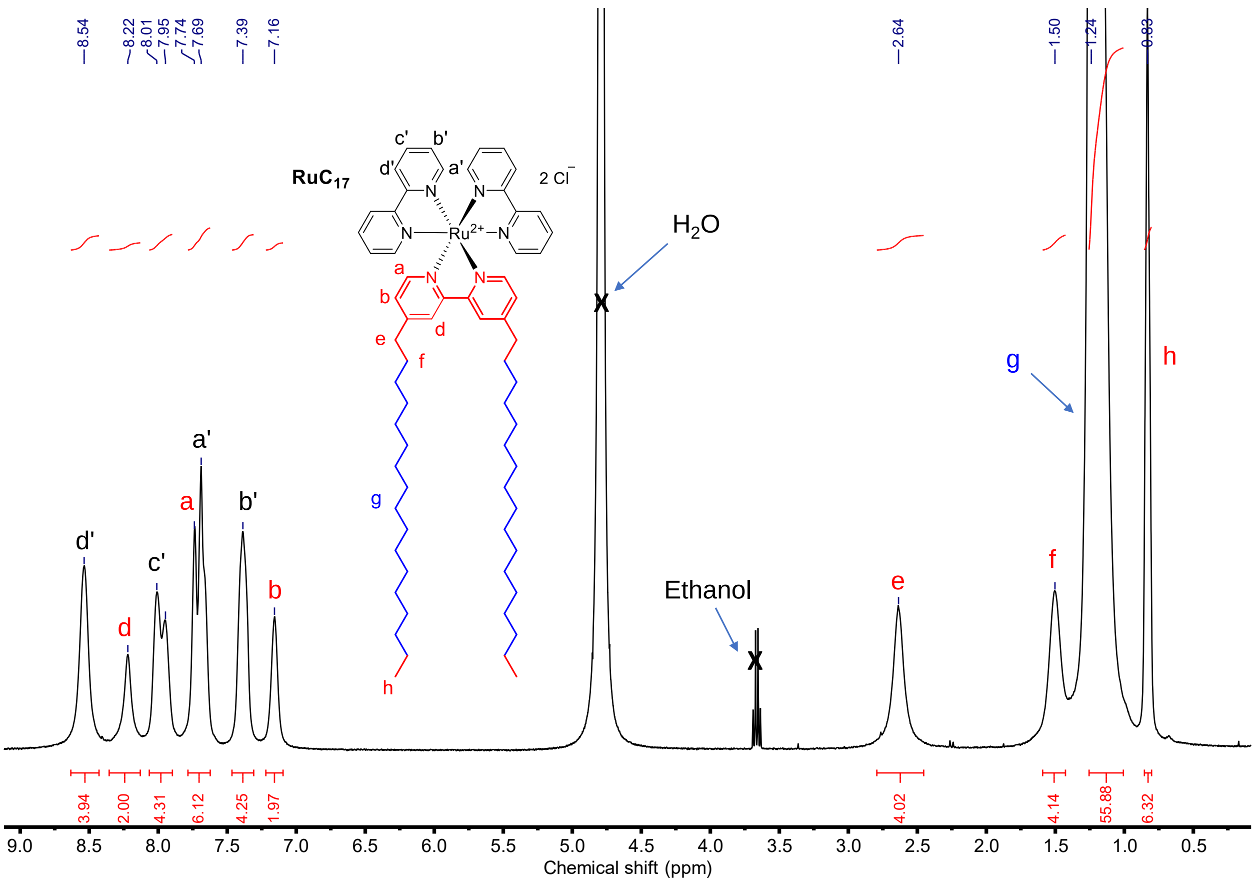

Figure S6: ^{1}H NMR spectrum of RuC_{17} in D_2O.

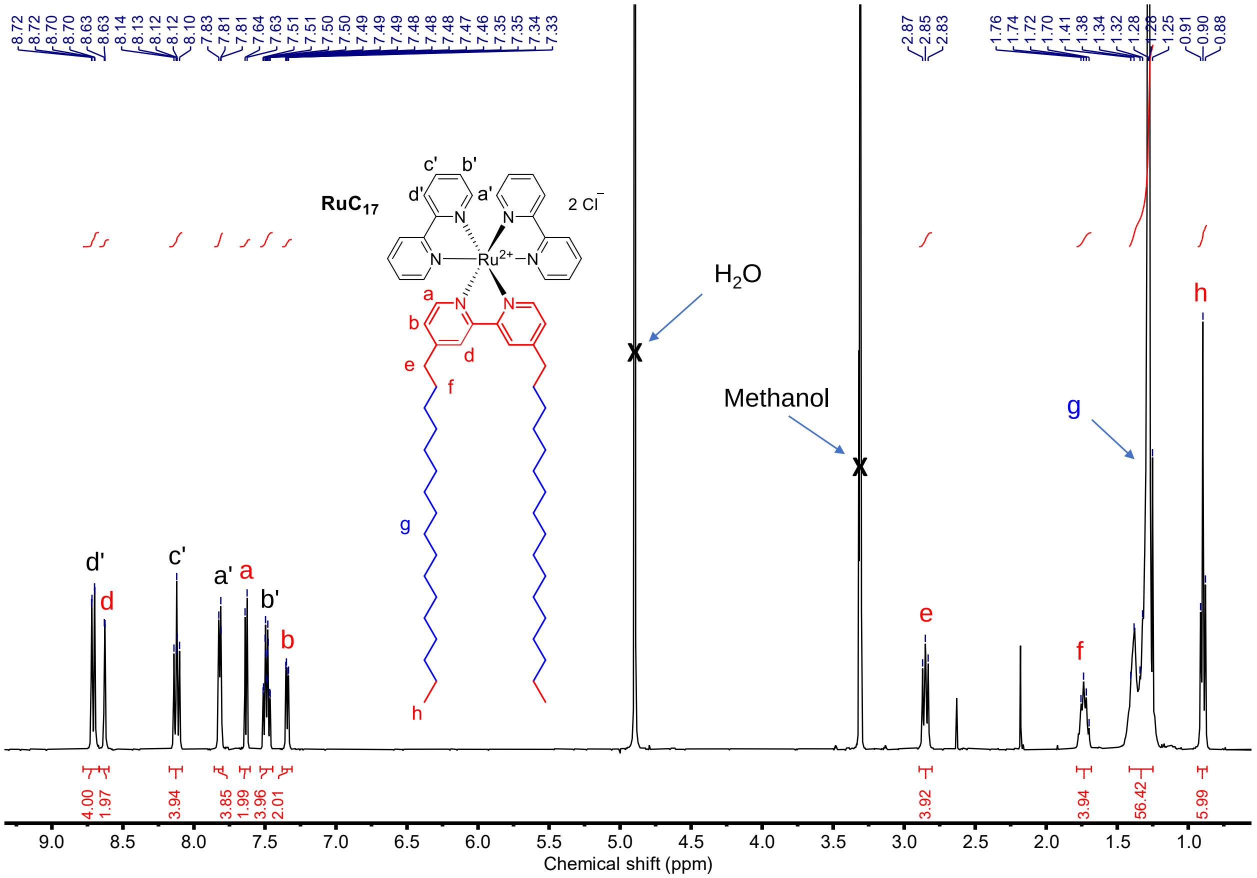

Figure S7: ^{1}H NMR spectrum of RuC_{17} in CD_3OD.

S3 UV−vis Absorption Spectra of Photosensitizers

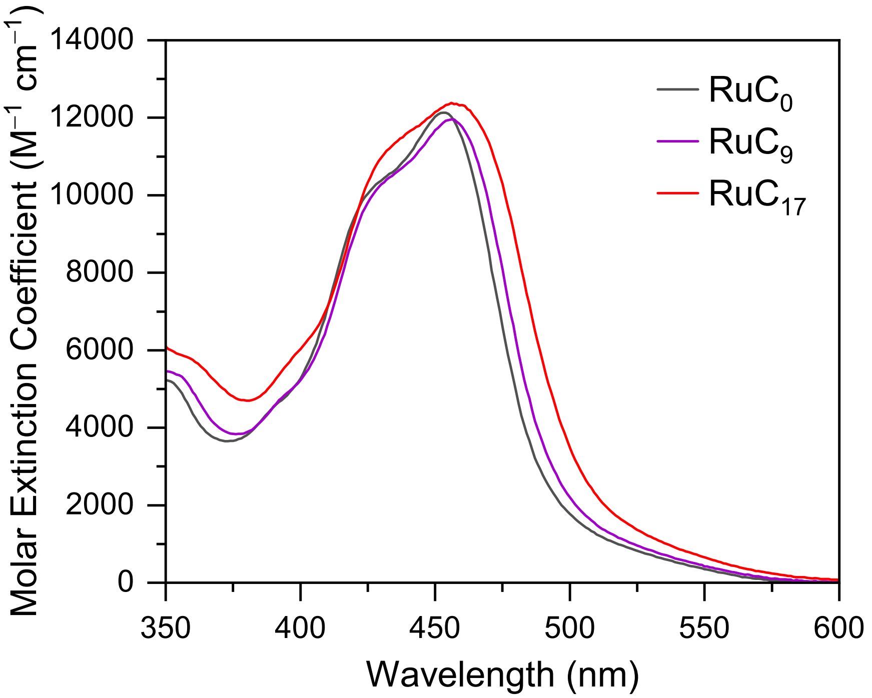

Figure S8: UV−vis absorption spectra of photosensitizers RuC$_0$, RuC$_9$ and RuC$_{17}$ in water.

S4 Estimation of Micelle Aggregation Numbers

The micelle aggregation number ($N_{\mathrm{aggregation}}$) is defined as the average number of surfactants required to form one spherical micelle. The value of $N_{\mathrm{aggregation}}$ depends on various factors such as the hydrocarbon tail length (L_{tail}), type of counter ion, surfactant concentration, and solution conditions (pH, ionic strength, temperature, additives, etc.). $N_{\mathrm{aggregation}}$ can be estimated by a predictive molecular thermodynamic approach that developed by Nagarajan and Ruckenstein as follows.[S9]

The volume of the hydrocarbon tail (V_{tail}) is determined by the number of carbon atoms within the tail (n_{c}):

$$V_{\mathrm{tail}} = V_{\mathrm{CH_3}} + (n_{\mathrm{c}} - 1)V_{\mathrm{CH_2}} \tag{1}$$

where $V_{\mathrm{CH_3}}$ and $V_{\mathrm{CH_2}}$ represent the volume of methyl group ($-CH_3$) and methylene group ($-CH_2$). At room temperature, $V_{\mathrm{CH_3}} = 54.6$ Å^3 and $V_{\mathrm{CH_2}} = 26.9$ Å^3.[S9]

The hydrocarbon tail length L_{tail} can be calculated by an empirical formula that developed by Tanford:[S10]

$$L_{\mathrm{tail}} = 1.50 + 1.26 n_{\mathrm{c}} \text{ Å} \tag{2}$$

Considering a spherical micelle with a radius $= L_{\mathrm{tail}}$, the total volume of one micelle (V_{micelle}) is determined by:

$$V_{\mathrm{micelle}} = \frac{4\pi L_{\mathrm{tail}}^3}{3} \tag{3}$$

Finally, the micelle aggregation number ($N_{\mathrm{aggregation}}$) can be estimated by the ratio of V_{micelle} to V_{tail}:

$$N_{\mathrm{aggregation}} = \frac{V_{\mathrm{micelle}}}{V_{\mathrm{tail}}} \tag{4}$$

As a result, $N_{\mathrm{aggregation}}$ for RuC_9 and RuC_{17} surfactants is estimated to be 33 and 104, respectively.

S5 Representative Steady-State Fluorescence Spectra

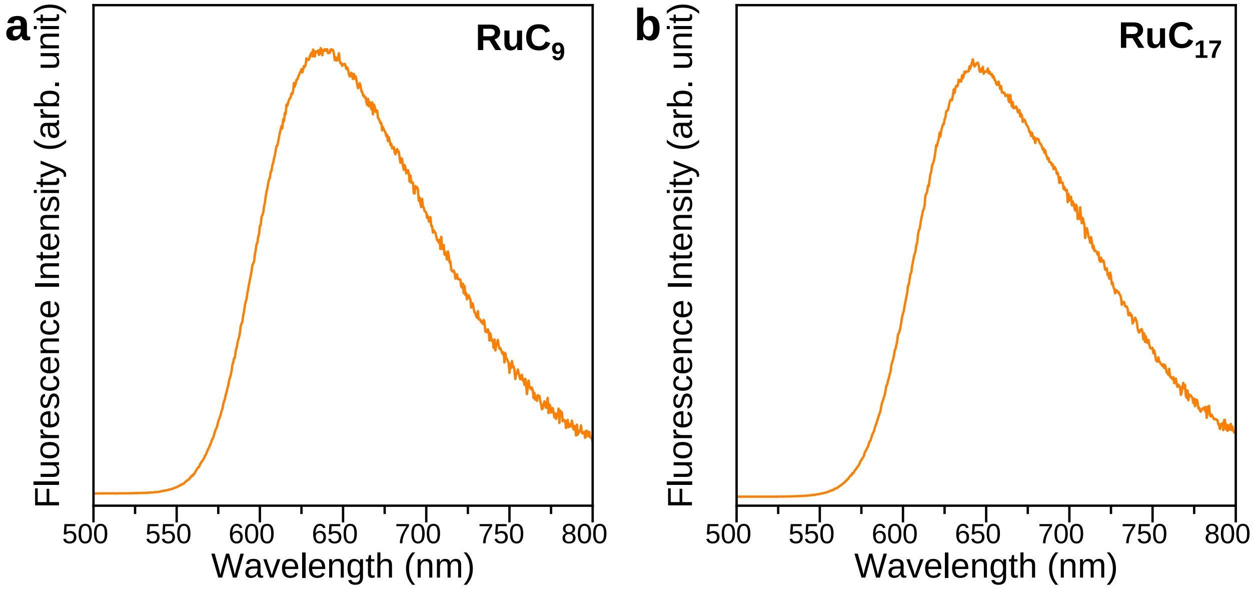

Figure S9: Representative steady-state fluorescence spectra for (a) RuC_9 and (b) RuC_{17}.

S6 Photocatalytic H_2 Production with RuC_9/RuC_{17} and [NiFeSe]-H_2ase

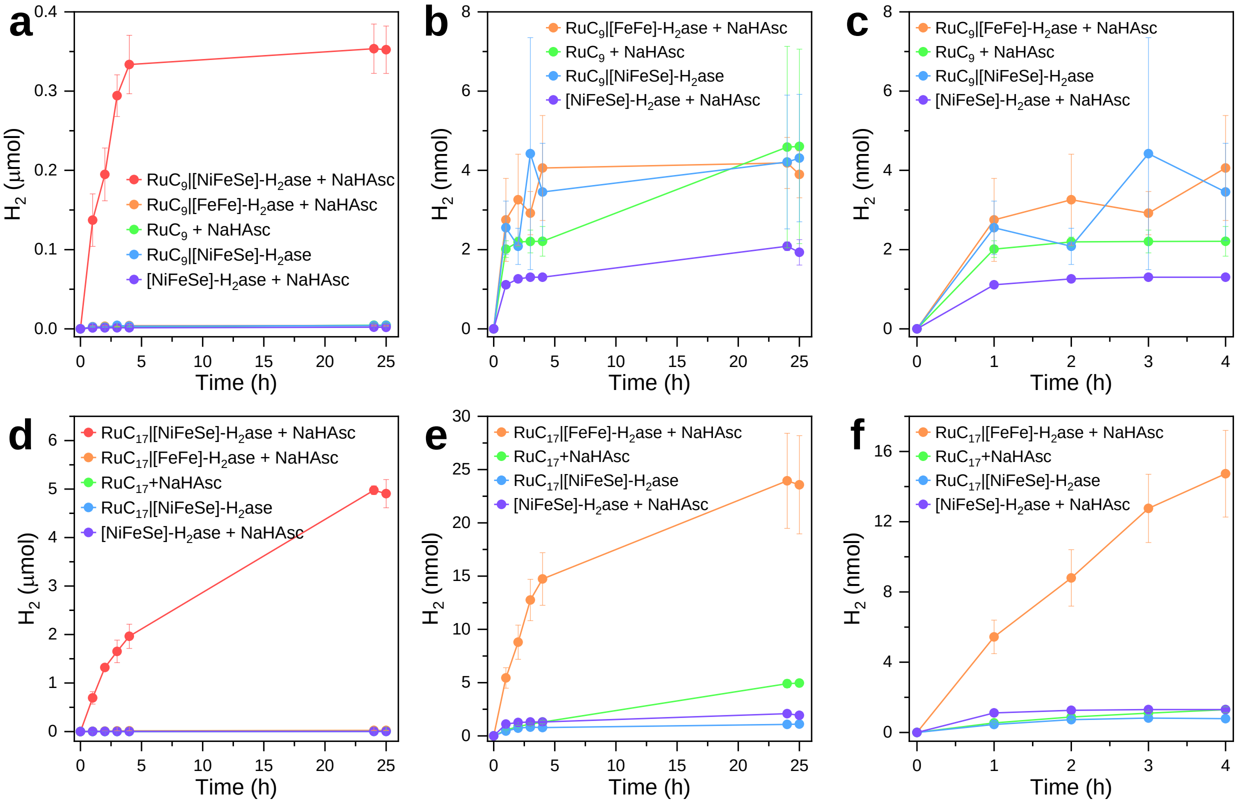

Figure S10: Time-dependent photocatalytic H_2 evolution of (a)-(c) 10 μM RuC_9 and (d)-(f) 5 μM RuC_{17} with various control experiments for 25 hours. (b), (c), (e), (f) are the magnification of (a), (d). Conditions: 0.5 mL anaerobic buffer containing 0.1 M MOPS, pH 7, AM 1.5G irradiation, 600 rpm stirring, 25°C. Error bars represent the standard deviation for a sample size of 3.

S7 Table of Photocatalytic H_2 Evolution using RuC_9

Table S1: Table of photocatalytic H_2 evolution using RuC_9.

Conditions	H_2 4 h (nmol)	TON 4 h	TOF 4 h (h^{-1})
RuC_9|[NiFeSe]-H_2ase+NaHAsc	333.6 ± 36.9	16680 ± 1845	4170 ± 461
RuC_9|[FeFe]-H_2ase+NaHAsc	4.1 ± 1.3	205 ± 65	51 ± 16
RuC_9+NaHAsc	2.2 ± 0.4	N.A.	N.A.
RuC_9|[NiFeSe]-H_2ase	3.5 ± 1.2	175 ± 60	44 ± 15
[NiFeSe]-H_2ase+NaHAsc	1.3 ± 0.1	65 ± 5	16 ± 1

S8 Table of Photocatalytic H_2 Evolution using RuC_{17}

Table S2: Table of photocatalytic H_2 evolution using RuC_{17}.

Conditions	H_2 4 h (nmol)	TON 4 h	TOF 4 h (h^{-1})
RuC_{17}|[NiFeSe]-H_2ase+NaHAsc	1964.4 ± 249.8	98220 ± 12490	24555 ± 3123
RuC_{17}|[FeFe]-H_2ase+NaHAsc	14.7 ± 2.5	735 ± 125	184 ± 31
RuC_{17}+NaHAsc	1.3 ± 0.2	N.A.	N.A.
RuC_{17}|[NiFeSe]-H_2ase	0.8 ± 0.1	40 ± 5	10 ± 1
[NiFeSe]-H_2ase+NaHAsc	1.3 ± 0.1	65 ± 5	16 ± 1

S9 Comparison among State-of-the-art Self-Assembled Photocatalytic Systems

Table S3: Comparison among state-of-the-art self-assembled photocatalytic systems combing amphiphilic photosensitizers and co-catalysts for H_2 evolution[S11–S14] and CO_2 reduction.[S5,S8,S15–S17]

Self-assembled photocatalysis	Product	Irradiation	TOF (h^{-1})	TON (time)	Selectivity (%)	Stability (h)	Yield (μmol h^{-1})	AQY (%)	Ref.
$Ru(bpy)_3$ micelle|[NiFeSe]-H_2ase	H_2	AM 1.5G	10417	250000 (24 h)	>99	<24	0.207	1.32	**This work**
$Ru(bpy)_3$ micelle|[NiFeSe]-H_2ase	H_2	AM 1.5G	24500	98000 (4 h)	>99	4	0.491	3.11	**This work**
micelle|[FeFe]-mimic	H_2	UV-light	77	539 (7 h)	>99	4	2.31	18.3	S11
$Ru(bpy)_3$ micelle|[FeFe]-mimic	H_2	450 nm LED	67	133 (2 h)	>99	2	NA	2.3	S12
$Ru(bpy)_3$ vesicle|[FeFe]-mimic	H_2	450 nm LED	67	67 (1 h)	>99	1	NA	NA	S13
$Ru(bpy)_3$ vesicle|[FeFe]-mimic	H_2	455 nm LED	39	59 (1.5 h)	>99	1.5	11.3	NA	S14
$Ru(bpy)_3$ micelle|[W]-FDH	$HCOO^-$	AM 1.5G	333	8000 (24 h)	>99	<24	0.006	0.04	**This work**
ZnMSA micelle|$CoTPPSO_3Na$	CH_4	AM 1.5G	9	6600 (30 d)	89	>720	0.026	5.6	S5
$Ru(bpy)_3$ liposome|CoP_L	CO	AM 1.5G	364	1456 (4 h)	77	4	0.023	0.16	S8
$Ru(bpy)_3$ micelle|CoP_L	CO	447 nm LED	1680	422 (15 min)	87	0.25	7.2	0.19	S15
$Ru(dtb)(bpy)_2$ vesicle|$Re(dtb)(CO)_3$	CO	AM 1.5G	13	190 (15 h)	98	15	3.5	0.38	S16
Re(dcbpy) micelle	CO	AM 1.5G	0.9	110 (120 h)	>99	120	0.138	0.76	S17

S10 Photocatalytic H_2 Production with RuC_0 and [NiFeSe]-H_2ase

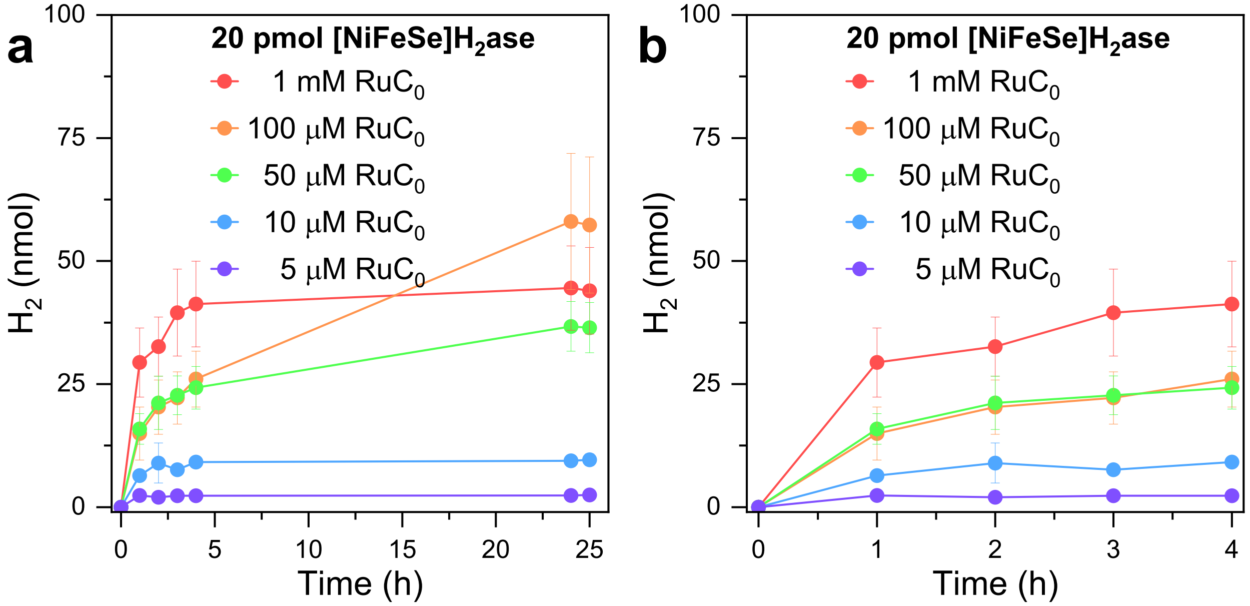

Figure S11: Time-dependent photocatalytic H_2 evolution with different concentrations of RuC_0 and 20 pmol [NiFeSe]-H_2ase. Conditions: 0.5 mL anaerobic buffer containing 0.1 M MOPS, 0.1 M NaHAsc, pH 7, AM 1.5G irradiation, 600 rpm stirring, 25°C. Error bars represent the standard deviation for a sample size of 3.

S11 Additional Transient Absorption and Emission

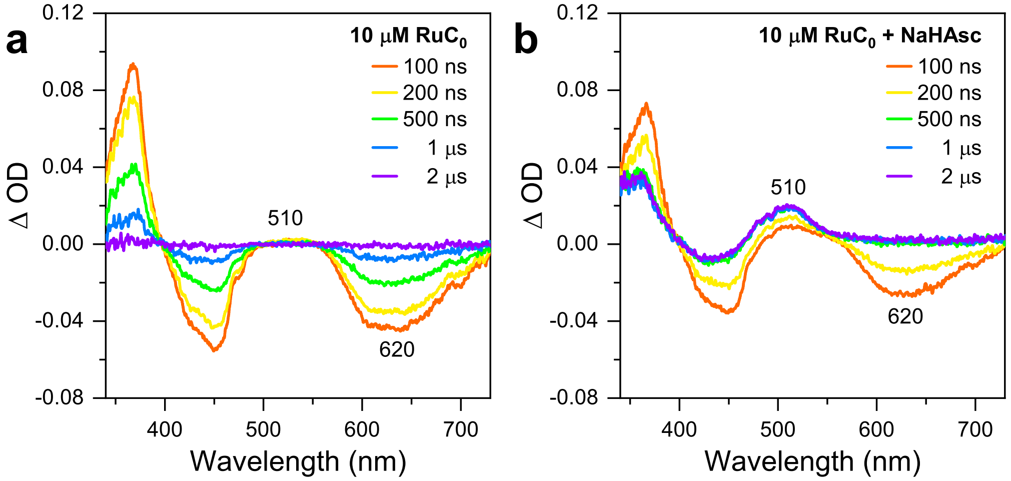

Figure S12: UV−vis transient absorption spectra of (a) 10 μM RuC_0 and (b) 10 μM RuC_0 + 0.1 M NaHAsc obtained following 460 nm laser excitation (10 ns, 7 mJ per pulse) in Ar-saturated 0.1 M MOPS buffer.

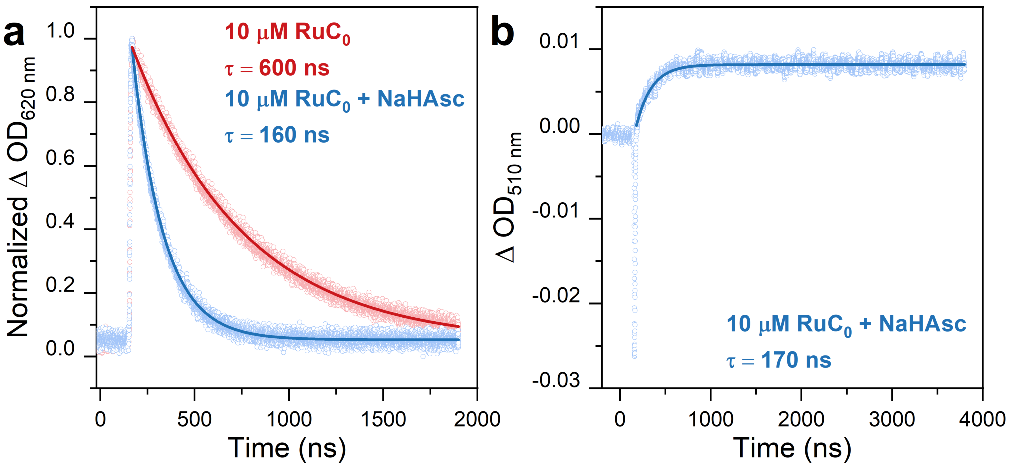

Figure S13: (a) Normalized time-resolved emission of 10 μM RuC_0 with and without NaHAsc. (b) The RuC_0 formation kinetic trace at 510 nm in the presence of 0.1 M NaHAsc. Condition: excitation wavelength at 460 nm (10 ns, 7 mJ per pulse) in Ar-saturated 0.1 M MOPS buffer.

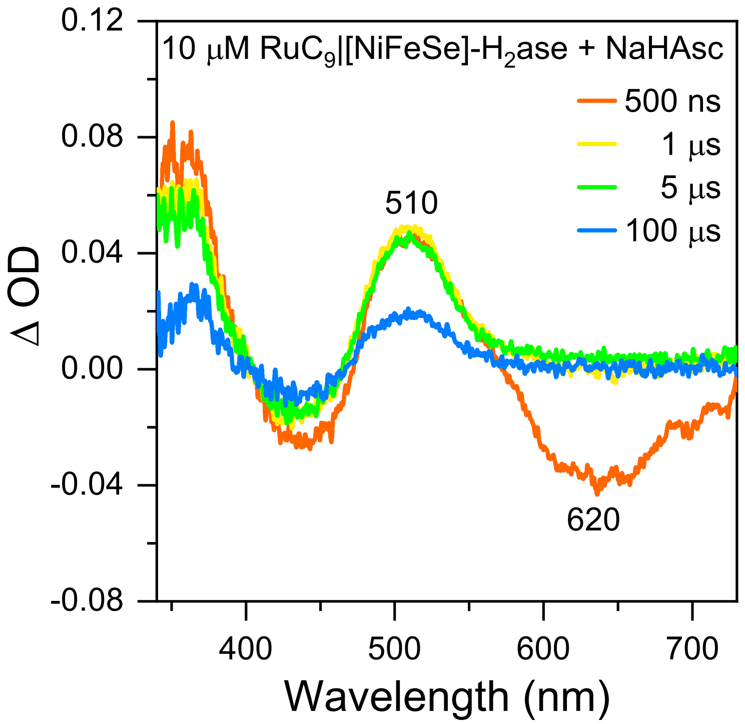

Figure S14: UV–vis transient absorption spectra of RuC_9 (10 μM) in the presence of NaHAsc (0.1 M) at different delayed time obtained following 460 nm laser excitation (10 ns, 7 mJ per pulse) in Ar-saturated aqueous MOPS (0.1 M) solution.

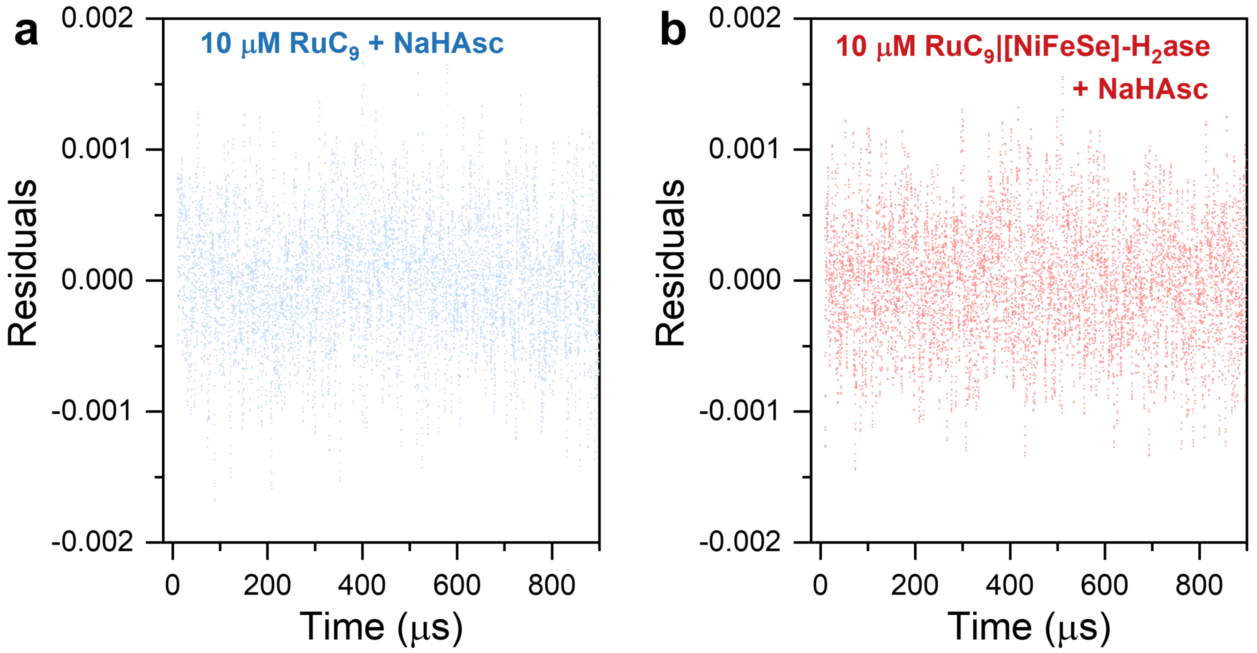

Figure S15: The corresponding fitting residuals for **Figure 3c**.

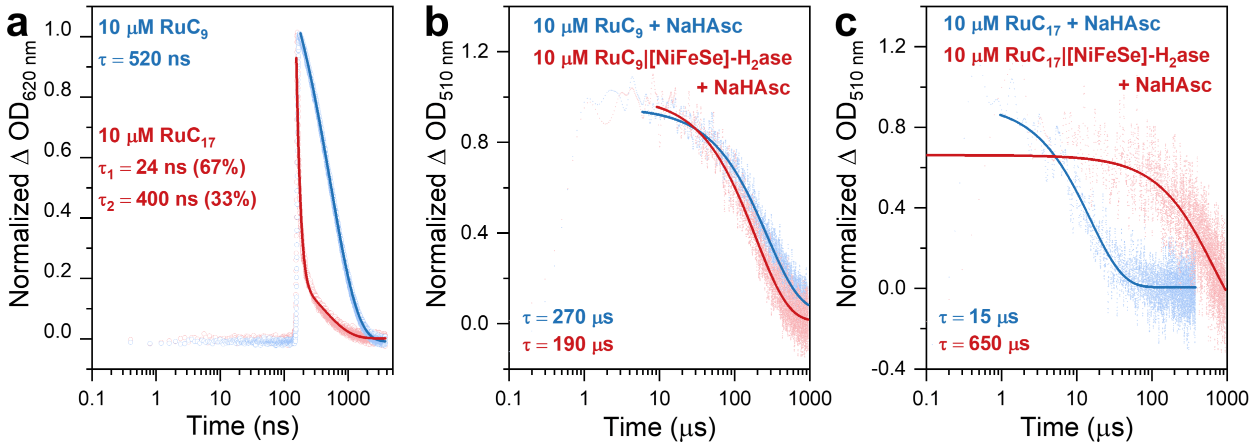

Figure S16: Corresponding Log plots for **Figure 3b–d**.

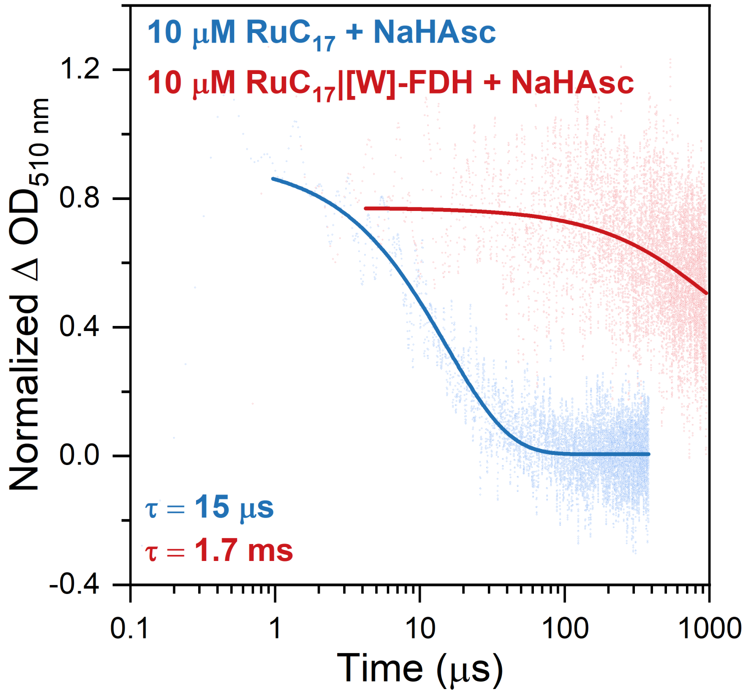

Figure S17: Corresponding Log plot for **Figure 4c**.

S12 Raw SDS-PAGE Gel Images

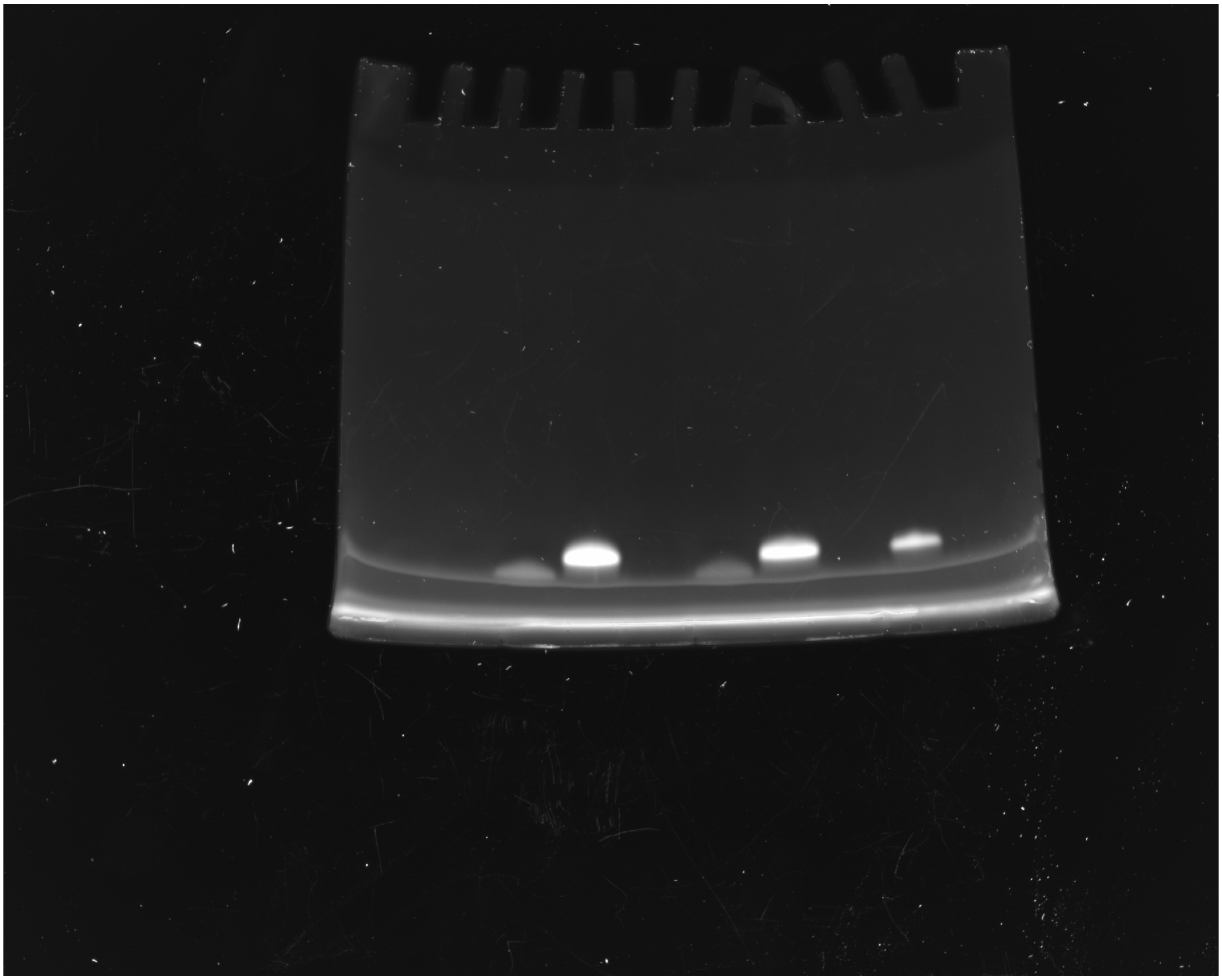

Figure S18: Raw SDS-PAGE gel image for **Figure 5b**.

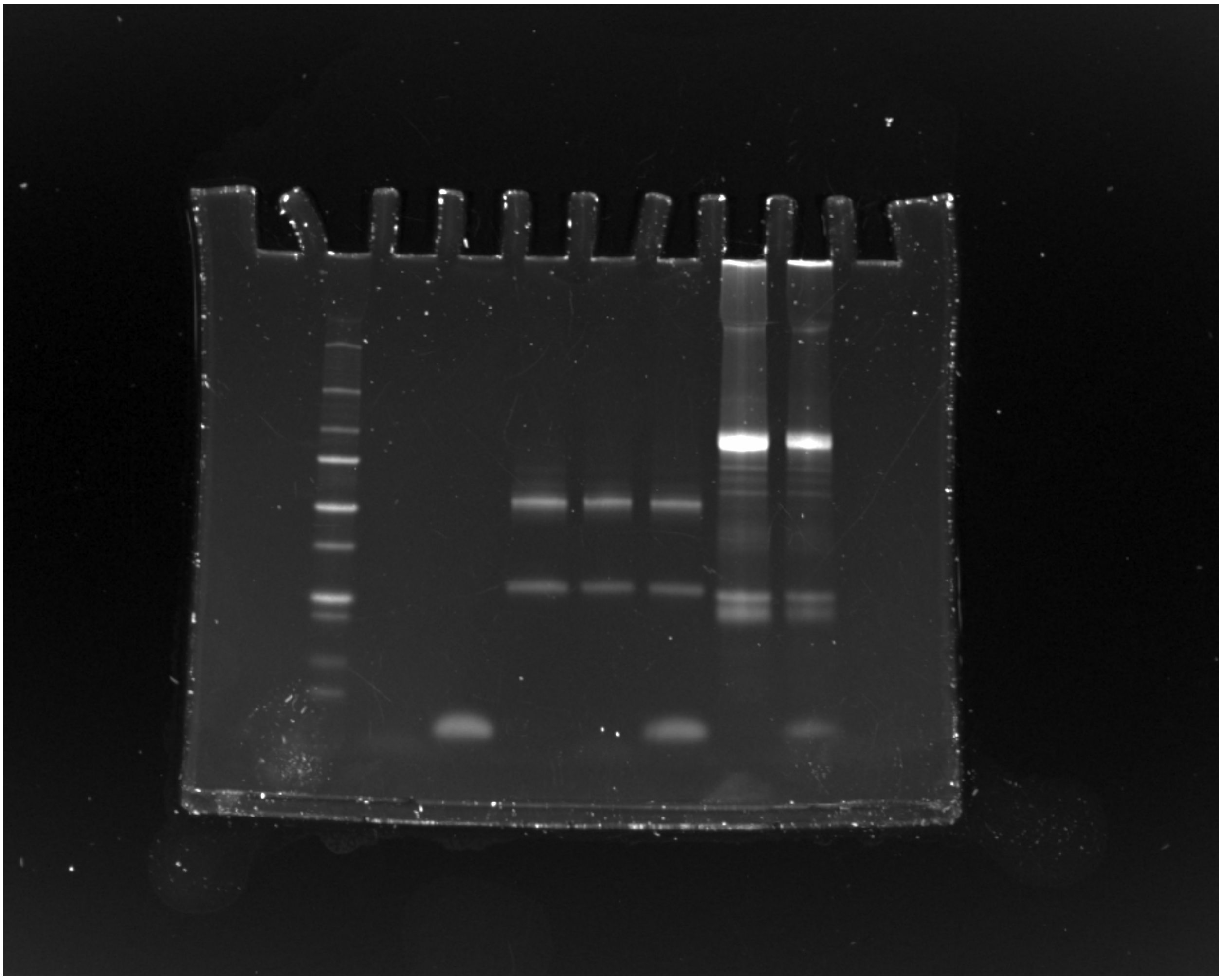

Figure S19: Raw SDS-PAGE gel image for **Figure 5c**.

References

(S1) Marques, M. C.; Tapia, C.; Gutiérrez-Sanz, O.; Ramos, A. R.; Keller, K. L.; Wall, J. D.; De Lacey, A. L.; Matias, P. M.; Pereira, I. A. The direct role of selenocysteine in [NiFeSe] hydrogenase maturation and catalysis. *Nat. Chem. Biol.* **2017**, *13*, 544–550.

(S2) Oliveira, A. R.; Mota, C.; Mourato, C.; Domingos, R. M.; Santos, M. F.; Gesto, D.; Guigliarelli, B.; Santos-Silva, T.; Romão, M. J.; Cardoso Pereira, I. A. Toward the mechanistic understanding of enzymatic CO_2 reduction. *ACS Catal.* **2020**, *10*, 3844–3856.

(S3) Liu, Y.; Webb, S.; Moreno-García, P.; Kulkarni, A.; Maroni, P.; Broekmann, P.; Milton, R. D. Facile functionalization of carbon electrodes for efficient electroenzymatic hydrogen production. *JACS Au* **2023**, *3*, 124–130.

(S4) Hatchard, C. G.; Parker, C. A. A new sensitive chemical actinometer-II. Potassium ferrioxalate as a standard chemical actinometer. *Proc. R. Soc. Lond. A* **1956**, *235*, 518–536.

(S5) Yu, J.; Huang, L.; Tang, Q.; Yu, S.-B.; Qi, Q.-Y.; Zhang, J.; Ma, D.; Lei, Y.; Su, J.; Song, Y.; Eloi, J.-C.; Harniman, R. L.; Borucu, U.; Zhang, L.; Zhu, M.; Tian, F.; Du, L.; Phillips, D. L.; Manners, I.; Ye, R.; Tian, J. Artificial spherical chromatophore nanomicelles for selective CO_2 reduction in water. *Nat. Catal.* **2023**, *6*, 464–475.

(S6) Rao, H.; Schmidt, L. C.; Bonin, J.; Robert, M. Visible-light-driven methane formation from CO_2 with a molecular iron catalyst. *Nature* **2017**, *548*, 74–77.

(S7) Timounay, Y.; Pannwitz, A.; Klein, D. M.; Biance, A.-L.; Hoefnagel, M. E.; Sen, I.; Cagna, A.; Le Merrer, M.; Bonnet, S. Interfacial Characterization of Ruthenium-Based Amphiphilic Photosensitizers. *Langmuir* **2022**, *38*, 9697–9707.

(S8) Rodríguez-Jiménez, S.; Song, H.; Lam, E.; Wright, D.; Pannwitz, A.; Bonke, S. A.; Baumberg, J. J.; Bonnet, S.; Hammarstrom, L.; Reisner, E. Self-assembled liposomes

enhance electron transfer for efficient photocatalytic CO_2 reduction. *J. Am. Chem. Soc.* **2022**, *144*, 9399–9412.

(S9) Nagarajan, R.; Ruckenstein, E. Theory of surfactant self-assembly: a predictive molecular thermodynamic approach. *Langmuir* **1991**, *7*, 2934–2969.

(S10) Tanford, C. *The hydrophobic effect: formation of micelles and biological membranes*; Wiley-Interscience, New York, 1980.

(S11) Goy, R.; Bertini, L.; Rudolph, T.; Lin, S.; Schulz, M.; Zampella, G.; Dietzek, B.; Schacher, F. H.; De Gioia, L.; Sakai, K.; Weigand, W. Photocatalytic hydrogen evolution driven by [FeFe] hydrogenase models tethered to fluorene and silafluorene sensitizers. *Chem. Eur. J.* **2017**, *23*, 334–345.

(S12) Wang, F.; Wen, M.; Feng, K.; Liang, W.-J.; Li, X.-B.; Chen, B.; Tung, C.-H.; Wu, L.-Z. Amphiphilic polymeric micelles as microreactors: improving the photocatalytic hydrogen production of the [FeFe]-hydrogenase mimic in water. *Chem. Commun.* **2016**, *52*, 457–460.

(S13) Becker, R.; Bouwens, T.; Schippers, E. C.; van Gelderen, T.; Hilbers, M.; Woutersen, S.; Reek, J. N. Photocatalytic Hydrogen Generation by Vesicle-Embedded [FeFe] Hydrogenase Mimics: A Mechanistic Study. *Chem. Eur. J.* **2019**, *25*, 13921–13929.

(S14) Troppmann, S.; Brandes, E.; Motschmann, H.; Li, F.; Wang, M.; Sun, L.; König, B. Enhanced Photocatalytic Hydrogen Production by Adsorption of an [FeFe]-Hydrogenase Subunit Mimic on Self-Assembled Membranes. *Eur. J. Inorg. Chem.* **2016**, *2016*, 554–560.

(S15) Bonke, S.; Trezza, G.; Bergamasco, L.; Song, H.; Rodríguez-Jiménez, S.; Hammarström, L.; Chiavazzo, E.; Reisner, E. Multi-Variable Multi-Metric Optimisation of Self-Assembled Photocatalytic CO_2 Reduction Performance using Machine Learning Algorithms. *J. Am. Chem. Soc.* **2024**, *146*, 15648–15658.

(S16) Ikuta, N.; Takizawa, S.-Y.; Murata, S. Photochemical reduction of CO_2 with ascorbate in aqueous solution using vesicles acting as photocatalysts. *Photochem. Photobiol. Sci.* **2014**, *13*, 691–702.

(S17) Ren, F.-Y.; Chen, K.; Qiu, L.-Q.; Chen, J.-M.; Darensbourg, D. J.; He, L.-N. Amphiphilic polycarbonate micellar rhenium catalysts for efficient photocatalytic CO_2 reduction in aqueous media. *Angew. Chem. Int. Ed.* **2022**, *61*, e202200751.
